# Prevalence of mental disorders in South Asia: A systematic review of reviews

**DOI:** 10.1017/gmh.2023.72

**Published:** 2023-11-13

**Authors:** Aishwarya L. Vidyasagaran, David McDaid, Mehreen R. Faisal, Muhammad Nasir, Krishna P. Muliyala, Sreekanth Thekkumkara, Judy Wright, Rumana Huque, Saumit Benkalkar, Najma Siddiqi

**Affiliations:** 1 Department of Health Sciences, University of York, Heslington, UK; 2 Care Policy and Evaluation Centre, Department of Health Policy, London School of Economics and Political Science, London, UK; 3 Department of Economics, Institute of Business Administration (IBA), Karachi, Pakistan; 4 Department of Psychiatry, National Institute of Mental Health & Neurosciences (NIMHANS), Bengaluru, India; 5 School of Health Sciences, University of Dundee, Dundee, UK; 6 Leeds Institute of Health Sciences, University of Leeds, Leeds, UK; 7 ARK Foundation, Dhaka, Bangladesh; 8 King’s College London, London, UK

**Keywords:** mental disorders, intentional self-harm, epidemiology, umbrella review, South Asia

## Abstract

Mental disorders are increasing in South Asia (SA), but their epidemiological burden is under-researched. We carried out a systematic umbrella review to estimate the prevalence of mental disorders and intentional self-harm in the region. Multiple databases were searched and systematic reviews reporting the prevalence of at least one mental disorder from countries in SA were included. Review data were narratively synthesised; primary studies of common mental disorders (CMDs) among adults were identified from a selected subset of reviews and pooled. We included 124 reviews. The majority (*n* = 65) reported on mood disorders, followed by anxiety disorders (*n* = 45). High prevalence of mental disorders and intentional self-harm was found in general adult and vulnerable populations. Two reviews met our pre-defined criteria for identifying primary studies of CMDs. Meta-analysis of 25 primary studies showed a pooled prevalence of 16.0% (95% CI = 11.0–22.0%, *I* ^2^ = 99.9%) for depression, 12.0% (5.0–21.0%, *I* ^2^ = 99.9%) for anxiety, and 14.0% (10.0–19.0, *I* ^2^ = 99.9%) for both among the general adult population; pooled estimates varied by country and assessment tool used. Overall, reviews suggest high prevalence for mental disorders in SA, but evidence is limited on conditions other than CMDs.

## Impact statement

Our umbrella review provides the most comprehensive estimates for the prevalence of mental disorders and intentional self-harm in South Asia (SA) and highlights that large proportions of the population in the region (both general-adult and specific vulnerable groups) are affected by these adverse health conditions. Evidence is critically lacking beyond common mental disorders on several conditions including schizophrenia and psychotic disorders, behavioural syndromes, personality disorders and intellectual disabilities. Although limited by heterogeneity and methodological quality of included studies, our review findings show an urgent need for countries in SA to formulate and implement clinical and policy measures for the prevention and early treatment of mental disorders and intentional self-harm. The pooled prevalence estimated for depression and anxiety in the general-adult population could serve as a reference for policymakers to take necessary action for curbing the growing burden of mental disorders in SA.

## Introduction

Mental disorders are recognised to be increasing globally, and contribute to a growing health, social and economic burden (World Health Organization, 2021). From 1990 to 2019, they have gone from the 13th to the 7th leading cause of disease burden in the world, with the number of disability-adjusted life-years (DALYs) due to mental disorders increasing from 80.8 million to 125.3 million; they also remain the second largest contributor to years lived with disability (GBD Mental Disorders Collaborators, 2022). Intentional self-harm accounts for a further 34.1 million DALYs, with their burden being greatest in low- and middle-income countries (Knipe et al., 2022). In South Asia (SA) (Afghanistan, Bangladesh, Bhutan, India, Maldives, Nepal, Pakistan and Sri Lanka) (The World Bank, 2019), rapid demographic and lifestyle changes are said to be associated with an exponential rise in mental and substance-use disorders, which health systems and services are unable to adequately meet (World Health Organization, 2016b; Ambekar et al., 2019). This has resulted in a considerable mental health treatment gap, with more than 75% of people affected in many countries not having access to the treatment they need (World Health Organization, 2016b; Gautham et al., 2020). Further, mental disorders have not been a policy priority among countries in the region, and their epidemiological and psychosocial burdens have been under-researched (Shidhaye et al., 2015). To address these issues and to improve the knowledge base for better planning and decision-making, an overall evaluation of the prevalence of mental disorders and intentional self-harm among countries in SA is needed.

Hossain et al. (2020) published an umbrella review, stating its advantages over a review of primary studies for understanding the population-level burden of mental disorders within the SA region. However, their inclusion criteria were limited to reviews solely conducted in SA (i.e., they excluded broader reviews, even if those reviews included some South Asian studies). We considered that expanding the scope of our umbrella review to identify all systematically conducted reviews, so long as they included evidence from at least one country in SA (whilst limiting our synthesis to South Asian studies), would provide a more complete picture of the prevalence of mental disorders and intentional self-harm in the region. In addition, a meta-analysis to provide an updated pooled estimate for the prevalence of mental disorders in the general adult population in SA would complement the overview provided by the umbrella review.

## Methods

The review was registered with PROSPERO (CRD42021282957) (McDaid et al., 2021). We followed the Joanna Briggs Institute (JBI) method for conducting the review (Aromataris et al., 2015) and the PRISMA guidelines for reporting (Page et al., 2021; Supplementary Appendix 1).

### Search strategy

We searched multiple electronic databases and research repositories, covering published and grey literature, on 29 September 2021 (Supplementary Appendix 2). Our searches included index terms, synonyms, and alternative phrases to cover mental disorders, South Asian countries, prevalence or epidemiology, and review types. We used the search strategies for ‘prevalence’ and ‘South Asia’ from Uphoff et al. (2019), and for ‘mental disorders’ from Mishu et al. (2021), adapting them to include all ICD-10 categories of mental disorders and intentional self-harm (World Health Organization, 2016a; Supplementary Appendix 3). Searches were developed by an information specialist (JW) and peer-reviewed by a second, using the PRESS checklist (McGowan et al., 2016). There were no limits for language or publication date. We also screened reference lists and forward citations of included studies. In addition, PROSPERO records were checked for any relevant ongoing or completed reviews. Retrieved records were de-duplicated in EndNote semi-automatically, using specified guidance (AUHE Information Specialists, 2016) and uploaded to COVIDENCE (www.covidence.org) for further evaluation.

### Inclusion criteria and study selection

We included systematic reviews (with or without meta-analyses) that searched two or more databases, and provided keyword and/or search strategies, as per the quality criteria of the AMSTAR2 checklist (Shea et al., 2017). Reviews reporting the prevalence or incidence of mental disorders in one or more countries in the World Bank-defined SA region were eligible. This included reviews that had data from countries beyond SA, but where we could extract the SA data on their own. All populations and settings were eligible, except studies of international military forces based in SA. Reviews on any mental, behavioural, and neurodevelopmental disorders (ICD-10, F-codes), or on suicide and intentional self-harm (ICD-10, X60-X84 codes) were eligible (Supplementary Appendix 4). Two authors independently evaluated all records at title and abstract and full-text screening stages. Discrepancies in screening were addressed through discussion with a third author.

### Data extraction and synthesis

A pre-piloted data extraction tool was uploaded to COVIDENCE. Two authors independently extracted data and performed quality appraisals for 10% of included reviews, with good agreement; discrepancies were identified and resolved through consensus. All remaining extractions were performed by a single author and checked by a second. Extraction items included objective and type of review, year of publication, name and timeframe of databases, originating countries of primary studies, sample size and characteristics, as well as reported prevalence or incidence of mental disorders. We used the AMSTAR2 tool for evaluating the methodological quality of included reviews (Shea et al., 2017).

Narrative synthesis was conducted according to the type of review (with or without meta-analysis) and mental disorders (ICD-10 categories), using tables and figures. For the reviews that went beyond SA, we only considered the pooled/range of estimates from the subgroup of studies that were relevant to the SA region. Next, we focused on the reviews with meta-analyses to summarise results for pooled prevalence of mental disorders in SA. Finally, to estimate prevalence for the common mental disorders (CMDs), depression and anxiety, we obtained data from primary studies in the included reviews. We limited this step to reviews with a pre-registered protocol (as a quality indicator), and those reporting on CMDs in the general adult population, given these conditions, which comprise the great majority of mental disorders, were the focus of the bulk of included reviews. Additional primary studies reporting CMD prevalence in SA were identified through forward citation screening of included reviews, to capture more recent studies.

Data extraction from primary studies was again performed by a single author and checked by a second on the following items: country, state or province of the study population, sample characteristics and sample size, and prevalence or incidence for each mental disorder. For quality assessment, we used the JBI Critical Appraisal Checklist for prevalence studies (Munn et al., 2014), but did not exclude ones at high risk of bias from further analysis. We created a ‘summary of findings’ table for primary studies and carried out meta-analyses using Stata (2007), Version 17.0 to produce a pooled estimate of prevalence for depression and anxiety among the general population in SA. Heterogeneity was assessed using *I* ^2^ statistics, and subgroup analyses based on country and outcome ascertainment tools were conducted to explore the sources. Evidence of publication bias was assessed using funnel plots and Egger’s test.

## Results

**Figure 1.**
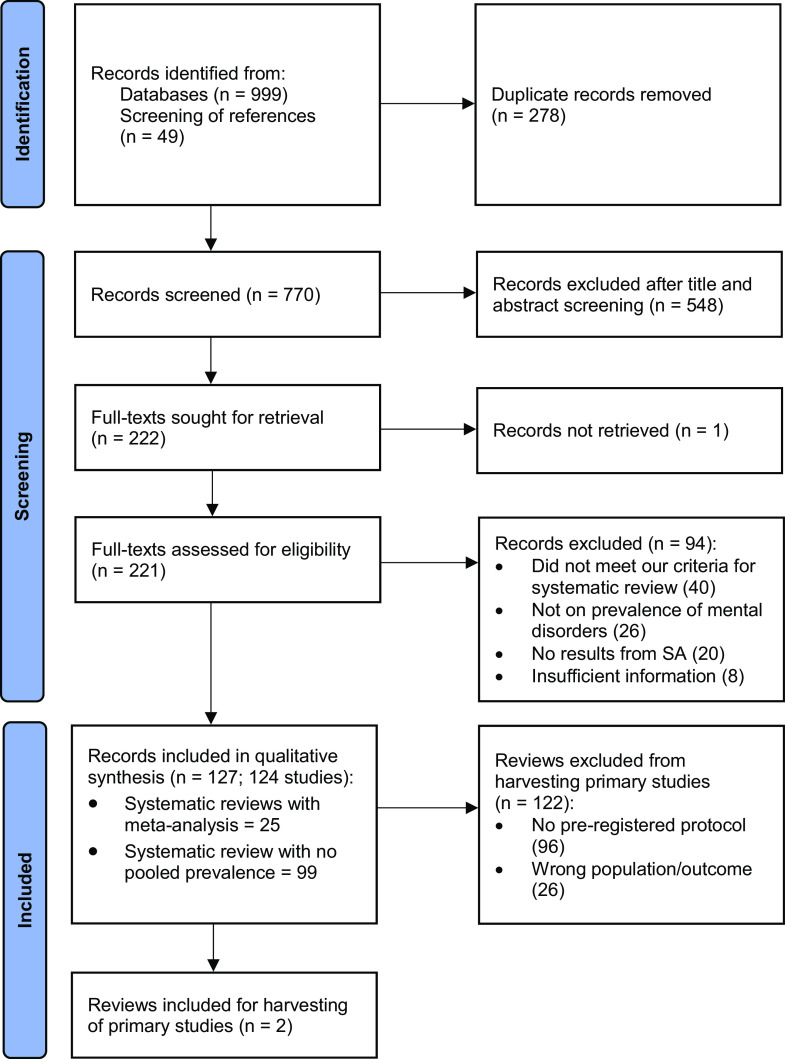
PRISMA flow chart for included reviews.

Our searches yielded 1,048 records, with 770 remaining after deduplication (Figure 1). Following title and abstract screening, 548 records were excluded, and all but one of the remaining 222 papers were obtained. Full-text screening resulted in the exclusion of 94 records (see Figure 1 and Supplementary Appendix 5 for details). 124 reviews (127 records) met our eligibility criteria and were included in the narrative synthesis. Three reports covering one review were merged (Barua et al., 2010; Barua et al., 2011a, 2011b); for another review, we merged and extracted data from both the original and updated reports (Oram et al., 2012; Ottisova et al., 2016).

For the meta-analyses of primary studies, we found only two reviews with pre-registered protocols, which reported on the prevalence of CMDs in the general adult population (Naveed et al., 2020; Zuberi et al., 2021). These provided 22 primary studies. Three additional studies were identified through forward citation screening of included reviews, resulting in 25 distinct primary studies for our meta-analyses (14 depression-only, three anxiety-only, and eight both) (see Supplementary Appendix 6 for flow chart of primary studies).

### Characteristics of reviews included in the review of reviews

Table 1 provides the summary characteristics of all included reviews. Twenty-five reviews had conducted meta-analyses providing pooled estimates for mental disorders in SA. A further 99 reviews did not provide pooled estimates, either because no meta-analysis was conducted (*n* = 61), no pooled values limited to SA countries were presented (*n* = 37), or pooled prevalence was not estimated (*n* = 1).

**Table 1.** Summary characteristics of included reviews

Reference	South Asian countries represented in the review (number of primary studies), sample size	Population	Mental disorder(s) and Prevalence/other measure of burden reported	AMSTAR-2 grading
Systematic reviews with meta-analysis (*n* = 25); reporting pooled prevalence (95% CI) unless otherwise specified				
Barua et al. (2010, 2011a, 2011b)	India (6); *n* = 2,499	Older people	Depression 21.9% (11.6–31.1)	Critically low
Steel et al. (2014)	Afghanistan (1), Bangladesh (1), India (7), Pakistan (3); *n* = 17,524	General adult population	CMD 19.8% (10.3–34.7)	Critically low
Cho et al. (2016)	India (2), Pakistan (1), Sri Lanka (1); *n* = 327	People with suicidal behaviour	Any mental disorder among fatal suicide 90.4% (71.8–97.2)	Low
Ranjan and Asthana (2017)	Afghanistan (1), Bangladesh (5), Bhutan (1), India (20), Nepal (3), Pakistan (3), Sri Lanka (1); *n* = 158,555	General adult population	Any mental disorder (rate per 1000) 122.0 (8.0–252.0)	Critically low
Upadhyay et al. (2017)	India (38); *n* = 20,043	Postpartum women	Depression 22.0% (19.0–25.0)	Low
Hussain et al. (2018)	India (37); *n* = 10,270	People with type 2 diabetes	Depression 38.0% (31.0–45.0)	Moderate
Chauhan et al. (2019)	India (4); *n* = 130,599	Children	ASD 0.1% (0.0–0.2)	Low
Hendrickson et al. (2019)	India (22); *n* = 5,122	Adults with AUD	Mood disorder 18.0% (5.6–45.1), Anxiety disorder 2.4% (0.9–5.8)	Low
Mahendran et al. (2019)	Bangladesh (3), India (12), Maldives (1), Nepal (1), Pakistan (14), Sri Lanka (2); *n* = 13,087	Pregnant women	Depression 24.3% (19.0–30.5)	Moderate
Pilania et al. (2019)	India (51); *n* = 22,005	Older people	Depression 34.4% (29.3–39.7)	High
Prabhu et al. (2019)	Bangladesh (1), India (12), Maldives (1), Nepal (5), Pakistan (9); *n* = 15,345	Postnatal women	Depression 26.0% (21.0–30.0)	Critically low
Uphoff et al. (2019)	Bangladesh (5), India (60), Pakistan (30), Multi-country (1); *n* = NR	Adults with NCD	Depression 41.0% (37.0–44.0), Anxiety 29.0% (22.0–36.0)	Moderate
Ganesan et al. (2020)	India (10); *n* = 6,513	Children and adolescents	Suicide attempt past-year 0.6% (0.0–1.8), lifetime 17.1% (5.0–35.4)	Low
Khan et al. (2020)	Pakistan (26); *n* = 7,652	University students	Depression 42.7% (34.8–50.9)	Moderate
Naveed et al. (2020)^a^	Bangladesh (8), India (81), Nepal (20), Pakistan (33), Sri Lanka (12), Multi-country (6); *n* = NR for all studies, range: 250 to 863,657	Adults – general, students, older people	CMD 28.4% (13.9–49.3), Alcohol abuse 12.9% (8.8–18.6), Opiates misuse 0.8% (0.2–2.5), Drug abuse 2.5% (0.1–32.1), Depression 26.4% (23.6–29.4), Bipolar 0.6% (0.3–1.0), Anxiety 25.8% (19.4–33.5), Panic disorder 1.3% (0.5–3.4), Phobias 1.8% (0.4–7.1), OCD 1.6% (0.4–5.5), PTSD 17.2% (11.0–25.9), Suicidal behaviour 6.4% (3.1–12.4)	Moderate
Abraham et al. (2021)	Pakistan (15); *n* = 2,890	HCWs	Depression 31.7% (18.7–48.3)	High
Assariparambil et al. (2021)	Bangladesh (7), India (89), Nepal (12), Pakistan (6), Sri Lanka (6); *n* = 65,060	Older people	Depression 42.0% (38.0–46.0)	Low
Atif et al. (2021)	Pakistan (43); *n* = 17,544	Perinatal women	Depression antenatal 37.0% (30.0–44.0), postnatal 30.0% (25.0–36.0)	Low
Choudhary et al. (2021)	India (20); *n* = 86,312	Older people	Dementia 2.0% (2.0–3.0)	Critically low
Hossain et al. (2021)	Bangladesh (7), India (19), Nepal (3), Pakistan (5), Sri Lanka (1); *n* = 41,402	General population and HCWs, COVID-19	Depression 34.1% (28.9–39.4), Anxiety 41.3% (34.7–48.1)	Low
Hosseinnejad et al. (2021)	Pakistan (6); *n* = 3,403	General population, after earthquakes	PTSD 49.2% (39.4–59.0)	Low
Kalra et al. (2021)	India (27); *n* = 7,880	Antenatal women	CMD 21.9% (17.5–26.3)	Moderate
Patra et al. (2021)	India (15); *n* = 1,617	Stroke survivors	Depression 55.0% (43.0–65.0)	Low
Yadav et al. (2021)	India (10); *n* = 2,362	Peri-menopausal women	Depression 42.5% (28.7–57.5)	High
Zuberi et al. (2021)^a^	Afghanistan (2), Pakistan (5); *n* = 19,314	General adult population	Afghanistan: SUD 0.0% (0.0–1.0), Depressive disorder 33.0% (7.0–75.0), Bipolar 0.0% (0.0–3.0), Anxiety disorder 25.0% (6.0–62.0), OCD 1.0% (0.0–5.0), Panic disorder 0.4% (0.1–2.0) PTSD 35.0% (4.0–87.0); Pakistan: SUD 32.0% (6.0–78.0), Depressive disorder 10.0% (4.0–25.0), Anxiety disorder 4.0% (0.0–27.0)	Moderate
Systematic reviews with no pooled estimates (*n* = 99), reporting prevalence/prevalence range unless otherwise specified				
Mirza and Jenkins (2004)	Pakistan (20); *n* = 9,170 for 17 relevant studies	General adult population	CMD 33.6%	Critically low
Mills et al. (2005)	India (5); *n* = 410	Tibetan refugee population	MDD 11.5–57.0%, Anxiety 25.0–77.0%, PTSD 11.0–23.0%	Low
Collins et al. (2006)	India (7), Nepal (1); *n* = 281	People with HIV/AIDS	Any mental disorder PWA 75.0% and HIVP 47.6%, Alcohol dependency 44.4%, Psychosis 5.0%, Depression 3.0–47.0%, Anxiety 25.0–36.0%, Adjustment disorder 27.8%, Suicidal intention/attempt 14.0%	Low
Lopes et al. (2007)	India (2); *n* = 2,603	Older people	Dementia 1.3–3.1%	Critically low
Mills et al. (2008)	Nepal (6); *n* = 4,712	Bhutanese refugee population	Depression 2.0%, Anxiety 4.0%, Phobia 18.5%, Dissociative disorder 8.0%, PTSD 25.0%, Somatoform pain 31.0%	Critically low
Klainin and Arthur (2009)	India (3), Nepal (1), Pakistan (3); *n* = 2,072	Postpartum women	Depression 4.9–56.0%	Critically low
Math and Srinivasaraju (2010)	India (16); *n* = 72,202	General adult population	Any disorder (rate per 1000) 9.5–102.0	Critically low
Das and Leibowitz (2011)	India (NR); *n* = NR	People with HIV/AIDS	Depression 33.0–70.0%, Anxiety 25.0–36.0%, Adjustment disorder 27.8%, Persistent suicidal intent/attempt 14.0%	Critically low
Maulik et al. (2011)	Bangladesh (2), India (1), Pakistan (2), Multi-country (1); *n* = 6,09,731	General population	Intellectual disability (rate per 1000) 0.9–156.0	Low
Fisher et al. (2012)	Bangladesh (4), India (4), Nepal (2), Pakistan (4); *n* = 5,126	Perinatal women	CMD antenatal 11.5–33.0% and postnatal 9.0–59.4%	Critically low
Hawton et al. (2013)	India (5); *n* = 649	Persons with self-harm	Depressive disorder 53.0–89.0%	Critically low
Jones and Coast (2013)	Bangladesh (1), India (3), Nepal (1), Pakistan (3); *n* = 2,479	Postpartum women	Depression 4.9–35.6%	Low
Nadkarni et al. (2013)	India (31); *n* = NR for all studies; range: 100 to 7,554	Over 50 years	AUD 1.1–70.0%	Critically low
Newman (2013)	Bangladesh (61); *n* = 12,021 for 16 relevant studies	General, 15 years and older	Depression 6.6–97.0%	Critically low
Rajapakse et al. (2013)	Sri Lanka (23); *n* = 74,482	General or clinical population	Intentional self-poisoning (rate per 100,000) 21.5–224.0	Low
Udina et al. (2013)	India (11), Sri Lanka (1); *n* = 799	Adult males	Dhat syndrome ~7% of patients seen at sexual health clinics; Depression 24.0–66.0%, Anxiety 13.0–37.0%	Critically low
Beckwith et al. (2014)	India (1), Pakistan (1); *n* = 16,318	Mental health outpatient	Personality disorder 1.0–60.0%	Critically low
de Bernier et al. (2014)	India (4); *n* = 5,616	General or clinical, adults	Personality disorders 1.3–52.0%	Critically low
Fuhr et al. (2014)	India (7), Nepal (1), Pakistan (1), Sri Lanka (2); *n* = NR	Perinatal women	Injury 1.1–17.9%, Suicide 1.0–10.7%	Moderate
Hossain et al. (2014)	Bangladesh (32); *n* = 25,767	General or clinical population	Any mental disorders 6.5–31.4%	Critically low
Jordans et al. (2014)	India (45), Bangladesh (26), Sri Lanka (18), Nepal (12), Pakistan (11), Afghanistan (1), Multi-country (1); *n* = NR	General or clinical population	Suicide (incidence per 100,000) 0.43–331.0	Moderate
Medlow et al. (2014)	India (1); *n* = 150	Homeless adolescents	Depression 8.0%	Critically low
Mendenhall et al. (2014)	Bangladesh (3), India (8), Pakistan (3); *n* = NR	People with type 2 diabetes	Depression 14.7–84.0%	Critically low
Pearson et al. (2014)	Sri Lanka (149); *n* = NR	General or clinical population	Suicide (rate per 100,000, as figure) ~25.0	Low
Rane and Nadkarni (2014)	India (36); *n* = NR	General or clinical population	Suicide (rate per 100,000) 82.0–95.0	Critically low
Aggarwal and Berk (2015)	India (27); *n* = 36,838	Adolescents	Depression 0.5–60.0%, GAD 13.0%, Social anxiety disorder 12.8%, PTSD 29.0%, Behavioural problems 1.8–24.7%, Suicidal behaviour 3.9–25.4%	Critically low
Malakouti et al. (2015)	Pakistan (2); *n* = 2,663	General population	Suicide (rate per 100,000) 0.6–1.1	Critically low
Norhayati et al. (2015)	Bangladesh (3), India (2), Nepal (2), Pakistan (2); *n* = 2,545	Postpartum women	Depression 3.1–59.4%	Critically low
Evagorou et al. (2016)	India (2), Nepal (1), Pakistan (1); *n* = 826	Postpartum women	Depression 4.9–63.0%	Critically low
McKenzie et al. (2016)	India (1); *n* = 70,302	General population	Intellectual disability (as figure) 1.0–1.2%	Low
Ottisova et al. (2016)	Nepal (1); *n* = 164	Victims of human trafficking	Depression 86.0%, Anxiety 90.2%, PTSD 13.4%	Moderate
Sahu et al. (2016)	India (12); *n* = 547	Amputees	Depression 10.4–63.0%, GAD 3.4–10.0%, PTSD 3.3–56.3%	Critically low
Tanzil Jamali (2016)	Pakistan (8); *n* = NR	Children and adolescents	Learning disability 24.8%, Emotional or behavioural disorders 34.0%	Critically low
Aggarwal et al. (2017)	India (2); *n* = 1,675	12–25 year olds	Non-suicidal self-harm 31.2%, Suicidal behaviour 6.1%, Suicide attempt 3.5%	Critically low
Ahmed et al. (2017)	Bangladesh (1), India (12), Sri Lanka (3); *n* = 3,024	People with suicidal behaviour	Depression among those who died by suicide 6.9–37.1%, attempted 20.7–59.7%	Low
Dennis et al. (2017)	Bangladesh (2); *n* = 1,394	Perinatal women	Anxiety 38.3%, Trait anxiety 29.4%	Low
Hossain et al. (2017)	Bangladesh (3), India (2), Sri Lanka (1); *n* = 41,620	Children and adolescents	ASD 0.1–1.1%	Critically low
Kuppili et al. (2017)	India (73); *n* = 16,073	Children and adolescents	ADHD 4.7–29.2%	Critically low
Naskar et al. (2017)	India (41); *n* = 34,119	People with type 1&2 diabetes	Depression 2.0–84.0%	Critically low
Salmanian et al. (2017)	Afghanistan (1); *n* = 1,011	Children and adolescents	Conduct disorder 4.8%	Low
Singh and Balhara (2017)	India (52); *n* = NR	People with cannabis use and psychiatric disorders	High frequency of psychiatric symptoms with SUDs, preponderance of cannabis-associated psychotic & affective disorders	Critically low
Woody et al. (2017)	India (3), Nepal (1), Sri Lanka (1); *n* = NR	Perinatal women	Depression NR for countries in SA	Critically low
Yatan Pal Singh et al. (2017)	India (13), Nepal (3); *n* = 51,008	General or clinical population	AUD 3.9–100%, Depression 2.7–94.3%	Critically low
Halim et al. (2018)	Bangladesh (3), India (2), Nepal (2), Pakistan (3); *n* = 4,546	Perinatal women	Depression antenatal 18–33% and postnatal 5–36%, Antenatal anxiety 29%, CMD 16–42%, Suicide attempts 2–5%	Critically low
Hunt et al. (2018)	India (1), Sri Lanka (1); *n* = 412	People with psychosis	AUD 3.0–11.0%, CUD 20.0%	Low
Jha et al. (2018)	Bangladesh (1), India (4), Pakistan (2), Sri Lanka (1); *n* = 3,323	Antenatal women	Depression 1.9–65.0%, Anxiety 26.0–49.0%	Low
Morina et al. (2018)	Nepal (2), Sri Lanka (2); *n* = 2,950	Refugee and IDP	Depression 22.0–80.0%, MDD 5.0–8.0%, Anxiety 33.0–81.0%, PTSD 3.0–53.0%	Critically low
Morina et al. (2018)	Afghanistan (1), India (1), Sri Lanka (1); *n* = 18,886	Civilian war survivors in area of conflict	Major depression 26.0–37.0%, PTSD 28.0–34.0%	Moderate
Shekhani et al. (2018)	Pakistan (110); *n* = NR for 2 relevant studies	General or clinical population	Suicide (incidence per 100,000) 0.43–2.86	Critically low
Shorey et al. (2018)	India (1), Nepal (1), Pakistan (3); *n* = 1,329	Postpartum women	Depression 5.0–62.0%	Moderate
Thapa et al. (2018)	Nepal (32); *n* = 4,152	Older people	Depressive disorders 4.4–53.2%, Anxiety 21.7–32.3%	Critically low
Arafat (2019)	Bangladesh (18); *n* = 14,942 for 3 relevant studies	General population	Suicide (rate per 100,000) 30.0–128.8	Critically low
Bhagavathula et al. (2019)	India (17), Pakistan (4); *n* = 4,441	People with hair dye poisoning	Suicide intent 75.0–99.9%	Critically low
Gilmoor et al. (2019)	India (56); *n* = 38,932	General or clinical population	PTSD 0.1–89.0%	Critically low
Knipe et al. (2019)	Bangladesh (2), India (28), Nepal (2), Pakistan (2), Sri Lanka (5); *n* = 9,888	People with suicidal behaviour	Any mental disorder among fatal suicide 48.0–96.0% and attempted 0.0–96.0%	High
Mytton et al. (2019)	Nepal (186); *n* = NR	General or clinical population	Self-harm NR for countries in SA	Critically low
Somrongthong et al. (2019)	India (8); *n* = 326 for 1 relevant study	Female sex workers 10–19 years	Suicidal attempts 41.0%	Critically low
Tay et al. (2019)	Bangladesh (1); *n* = 148	Rohingya refugee population	Depression 89.0%, PTSD 36.0%	Critically low
Vaidyanathan et al. (2019)	India (39); *n* = 6,663	General or clinical population	Probable ED 4.0–45.4%, ED 1.25%	Critically low
Abate et al. (2020)^a^	India (3), Pakistan (3); *n* = 1,312	People undergoing surgery with anaesthesia	Preoperative anxiety 24.0–88.0%	Moderate
Akhtar et al. (2020)	India (1), Nepal (2), Pakistan (1), Sri Lanka (1); *n* = 7,495	University students	Depression 9.3–53.1%	Moderate
Banerjee et al. (2020)	Bangladesh (1), India (11), Pakistan (1); *n* = 7,936	General or clinical, COVID-19	Depression 10.5–34.9%, Anxiety 38.2–39.5%	Critically low
Blackmore et al. (2020)	Nepal (1); *n* = 574	Adult refugees	Depression 1.9%, Anxiety 4.7%, PTSD 26.8%	Moderate
Devarapalli et al. (2020)	India (32); *n* = NR for all studies; range: 103 to 114,068	Tribal population	Depression 8.3%, Anxiety 6.4%, Adjustment disorder 9.0%, Somatoform pain 14.0%, PTSD 9.6%, Alcohol abuse 36.2%, Binge eating 6.4%, Bulimia nervosa 1.4%, Self-harm 11.2%, Suicide 14.2%	Critically low
Dua and Grover (2020)	India (33); *n* = 13,227	Clinical population (liaison psychiatry settings)	Delirium 2.8–43.4%, Dementia 0.9–3.8%, SUD 1.8–28.9%, Organic psychosis 0.6–25.5%, Psychotic illness 3.2–33.3%, Depression 1.5–24.4%, Bipolar 2.3–10.4%, Anxiety 1.1–13.1%, Adjustment 0.4–16.0%, Dissociation 0.9–8.3%, Psychosomatic 0.8–7.7%, Psychosexual 0.7%, Personality disorder 0.6–5.3%, Mental retardation 0.6–7.0%, Conduct disorder 0.8%, ADHD 0.4–0.8%, Self-harm 2.7–33.9%	Critically low
Fekadu Dadi et al. (2020)	NR; *n* = NR	Antenatal women	Depression NR for countries in SA	Moderate
Gilan et al. (2020)	India (1); *n* = 662	General or clinical, COVID-19	Hypochondriac fear 37.8%	Low
Hunt et al. (2020)	Sri Lanka (1); *n* = 109	People with MDD	AUD 21.1%, CUD 1.8%	Critically low
Janse Van Rensburg et al. (2020)	India (11), Pakistan (3), Sri Lanka (1), Multi-country (1); *n* = NR	People with tuberculosis	AUD 4.0–58.0%, Depression 8.5–84.0%, Anxiety 2.0–47.2%,	Critically low
Kalra et al. (2020)	Bangladesh (8), India (24), Nepal (3), Pakistan (7); *n* = 12,650	Adults with type 2 diabetes	Depression 11.6–67.5%	Critically low
Karimi et al. (2020)	India (1); *n* = 133	People with migraine	Anxiety 16.54%	Low
Khunsa Junaid (2020)	India (2); *n* = 8,484	HCWs, COVID-19	Depression 34.8%	Low
Lasheras et al. (2020)	India (1); *n* = 250	Medical students, COVID-19	Anxiety 17.2%	Low
Liu et al. (2020)	Sri Lanka (1); *n* = 335	People with history of deliberate self-harm	Non-fatal repetition of self-harm (incidence) 3.0%	High
Qiu et al. (2020)	Bangladesh (1), India (1), Nepal (2); *n* = 43,401	Children and adolescents	ASD 0.1–0.3%	Low
Rahele et al. (2020)	Pakistan (1), Sri Lanka (1); *n* = 1,786	Perinatal women, COVID-19	CMD 14.3%, Depression 19.5%, Anxiety 17.5%	Critically low
Winsper et al. (2020)	Bangladesh (1); *n* = 766	12–18-year-olds	Personality disorder 0.5%	High
Yan et al. (2020)	Sri Lanka (1); *n* = 257	Perinatal women, COVID-19	Depression 28.0%, Anxiety 26.0%	Moderate
Al Falasi et al. (2021)	India (1); *n* = 426	HCWs, COVID-19	PTSD 7.3%	Low
Al Mamun et al. (2021)	Bangladesh (9); *n* = 18,201	General or clinical, COVID-19	Suicidal behaviour 6.1%	Critically low
Amiri and Behnezhad (2021)	Bangladesh (1), India (2), Nepal (1); *n* = 1,034	Postpartum women	Suicide attempt 4.0–18.0%	Critically low
David Franciole de Oliveira et al. (2021)	India (1); *n* = 100	Teachers, COVID-19	CMD NR for countries in SA	Low
Dong et al. (2021)	India (1); *n* = 50	People with COVID-19	Depression 24.0%, Anxiety 32.0%	Moderate
Dutta et al. (2021)	India (4), Nepal (1), Pakistan (2); *n* = 1,869	HCWs, COVID-19	Depression 28.2–72.3%, Anxiety 34.0–85.7%	Moderate
Fellmeth et al. (2021)	India (7); *n* = 1,003	Perinatal women	Depression 12.5–18.0%	High
Ghazanfarpour et al. (2021)	Pakistan (1), Sri Lanka (1); *n* = 1,786	Pregnant women, COVID-19	Depression 19.5%, Anxiety 14.3–17.5%	Critically low
Hosen et al. (2021)	Bangladesh (24); *n* = 49,806	General or clinical, COVID-19	Depression 12.1–82.4%, Anxiety 10.6–81.8%, PTSD/Stress 11.1–85.6%	Critically low
Jephtha and Jagadeesan (2021)	India (1); *n* = 15,981	HCWs, COVID-19	CMD NR for countries in SA	Critically low
Kar et al. (2021)^a^	Bangladesh (1) India (5) Pakistan (2); *n* = NR	Adult males	Dhat syndrome 64.6%	Critically low
Liu et al. (2021)	India (1); *n* = 662	General, COVID-19	Anxiety 58.5%	Moderate
Mahadevan et al. (2021)	Bangladesh (1), India (11), Sri Lanka (1); *n* = 2,013	Stroke survivors	Depression 13.8–100.0%, Anxiety 80.9%	Moderate
Mahmud et al. (2021)	Bangladesh (2), India (7), Nepal (1), Pakistan (3); *n* = 5,422	HCWs, COVID-19	Depression 37.5–53.6%, Anxiety 41.9–62.2%	Moderate
Mamun (2021)	Bangladesh (7); *n* = 21,534	Students, COVID-19	Depression 46.9–82.4%, Anxiety 26.6–96.8%	Critically low
Mohammadi et al. (2021)	India (3), Sri Lanka (1); *n* = 3,757	Children and adolescents	Conduct disorder 1.0–7.0%	High
Necho et al. (2021)	India (1); *n* = 662	General adult, COVID-19	CMD NR for countries in SA	Low
Panda et al. (2021)	Bangladesh (1), India (1); *n* = 505	Children, adolescents and caregivers, COVID-19	Anxiety, depression and/or sleep disturbance 57.0–68.0%	Low
Santabárbara et al. (2021)^a^	Bangladesh (2), India (2); *n* = 4,092	General, COVID-19	Anxiety 28.0–43.0%	Moderate
Vanderkruik et al. (2021)	Bangladesh (4); *n* = NR	Adolescents	Depression during pregnancy 7.0–14.0% and postpartum 10.4–36.2%	Moderate
Wang et al. (2021)	Bangladesh (2); *n* = NR	College students, COVID-19	Depression 47.0–82.0%, Anxiety 33.0–84.0%	Critically low

Abbreviations: ADHD, attention deficit hyperactivity disorder; ASD, autism spectrum disorder; AUD, alcohol use disorder; CI, confidence interval; CMD, common mental disorder; CUD, cannabis use disorder; ED, eating disorder; GAD, generalised anxiety disorder; HCW, health care worker; HIVP, HIV positive; IDP, internally displaced population; MDD, major depressive disorder; NCD, non-communicable disease; NR, not reported; OCD, obsessive compulsive disorder; PTSD, post-traumatic stress disorder; PWA, people with AIDS; SA, South Asia; SUD, substance use disorder.

a Reporting discrepancy noted.

The earliest review was published in 2004 (Mirza and Jenkins, 2004), with the majority (*n* = 116) published after 2010. The number of databases searched ranged from two to fourteen, and the majority of reviews presented evidence from India (*n* = 90). The number of South Asian primary studies ranged from one to 149, and sample size ranged from 109 to 863,657 (not reported in 15 reviews). Reviews covered diverse populations, with participants recruited from a range of clinical and community-based settings. Only ten reviews were rated as ‘high’ quality, while most (61) were rated as ‘critically low’ (details in Supplementary Appendix 7).

A total of 65 reviews presented the prevalence of mood (affective) disorders including depressive and bipolar disorders, followed by 45 on anxiety disorders, and 10 on a combination of mood and anxiety disorders, grouped together as CMDs. A further nine reviews reported the prevalence of substance use disorders (SUDs), while others covered a range of other mental disorders: seven on behavioural and emotional disorders with usual onset in childhood and adolescence, including conduct disorder and attention-deficit hyperactivity disorder (ADHD), four on pervasive developmental disorders including autism spectrum disorder (ASD), three each on dementia, schizophrenia and psychotic disorder, personality disorder, and intellectual disabilities, and two on eating disorders. Of these, only one included a meta-analysis providing a pooled estimate of ASD prevalence among children in India. We also found six reviews that reported the prevalence of ‘any mental disorder’ and 23 that reported on suicide and intentional self-harm. Many identified reviews covered mental disorders in specific population subgroups including older people, perinatal women, students, healthcare workers (HCWs), and persons with comorbidities. Twenty-two reviews focused on the impact of COVID-19 on the psychosocial health of various population groups (Supplementary Appendix 8).

### Summary of pooled prevalence from systematic reviews with meta-analysis

We now focus on the 25 reviews with meta-analyses on the prevalence of various mental disorders in SA. Eleven were exclusively of studies conducted in India, four in Pakistan, and the remaining 10 covered multiple countries in the region. The population comprised all adults (including perinatal women and older people, *n* = 1), general adults (*n* = 3), adults with specific conditions such as alcohol use disorders (AUD) or non-communicable disease (NCD) (*n* = 6), women (*n* = 6), older people (*n* = 4), children and adolescents (*n* = 2), HCWs (*n* = 2), and university students (*n* = 1). In general, these reviews reported high pooled prevalence of mental disorders among both general-adult (up to 33.0% for depression) (Naveed et al., 2020), and specific population subgroups (up to 55.0% for depression among stroke survivors) (Patra et al., 2021). The pooled prevalence of suicidal behaviours among adults was 6.4% (95% CI = 3.1–12.4) (Naveed et al., 2020), and among children and adolescents was 17.1% (5.0–35.4) (Ganesan et al., 2020), whereas the pooled prevalence of any mental disorder among victims of suicide was 90.4% (71.8–97.2) (Cho et al., 2016).

**Figure 2.**
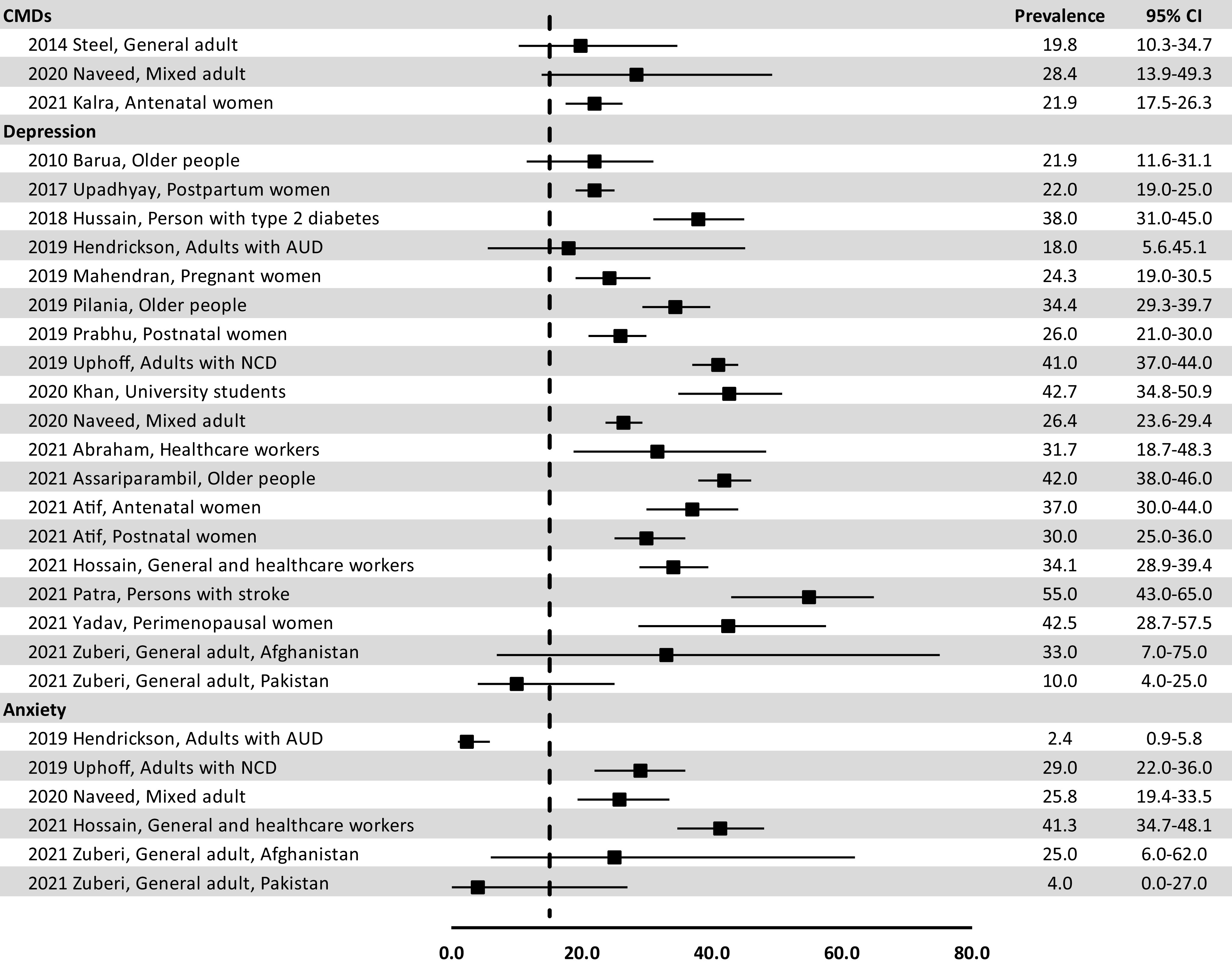
Pooled estimates of CMDs, depression and anxiety from meta-analytic reviews. *Note:* Two studies (2021 Atif and 2021 Zuberi) provided two relevant estimates each for different population groups; the vertical dotted line denotes a pooled prevalence of 14.0% (drawn to correspond with the Figure 3 forest plot).

We identified 19 pooled estimates for mood disorders (17 studies), followed by six for anxiety disorders (5 studies), and three for CMDs (Figure 2). The pooled prevalence (range) for depressive disorders in the general population was 10.0% (4.0–25.0) to 33.0% (7.0–75.0). Estimates were generally higher for specific population subgroups, including older people (21.9% (11.6–31.1) to 42.0% (38.0–46.0)), perinatal women (22.0% (19.0–25.0) to 37.0% (30.0–44.0)), peri-menopausal women (42.5% (28.7–57.5)), university students (42.7% (34.8–50.9), HCWs = 31.7% (18.7–48.3) to 34.1% (28.9–39.4)), and adults with comorbidities (18.0% (5.6–45.1) to 55.0% (43.0–65.0)). Similarly, pooled prevalence (range) for anxiety disorders in the general population was 4.0% (0.0–27.0) to 25.8% (19.4–33.5); for adults with comorbidities, it was 2.4% (0.9–5.8) to 29.0% (22.0–36.0), and among all adults and HCWs during the COVID-19 pandemic it was 41.3% (34.7–48.1). Based on reviews covering multiple countries in SA, the pooled prevalence of CMDs in the general adult population alone was estimated to be 19.8% (10.3–34.7) (Steel et al., 2014), whereas it was higher (28.4% (13.9–49.3)) among adult populations that included older people and perinatal women (Naveed et al., 2020). One review from India reported a pooled value of 21.9% (17.5–26.3) for prevalence of CMDs among antenatal women (Kalra et al., 2021).

We also found one general-adult, population-based estimate for the pooled prevalence of any mental disorder, covering all countries in SA except Maldives and presented as a rate per 1000 (95% CI): 122.0 (8.0–252.0) (Ranjan and Asthana, 2017). In addition, we found two meta-analyses reporting SUDs prevalence of 0.0% (0.0–1.0) to 32.0% (6.0–78.0) (Naveed et al., 2020; Zuberi et al., 2021), one on dementia prevalence (2.0% (2.0–3.0)) (Choudhary et al., 2021) and one on ASD prevalence (0.1% (0.0–0.2)) (Chauhan et al., 2019). These are not presented in Figure 2.

### Pooled prevalence of depression and anxiety in the general adult population from primary studies

**Figure 3.**
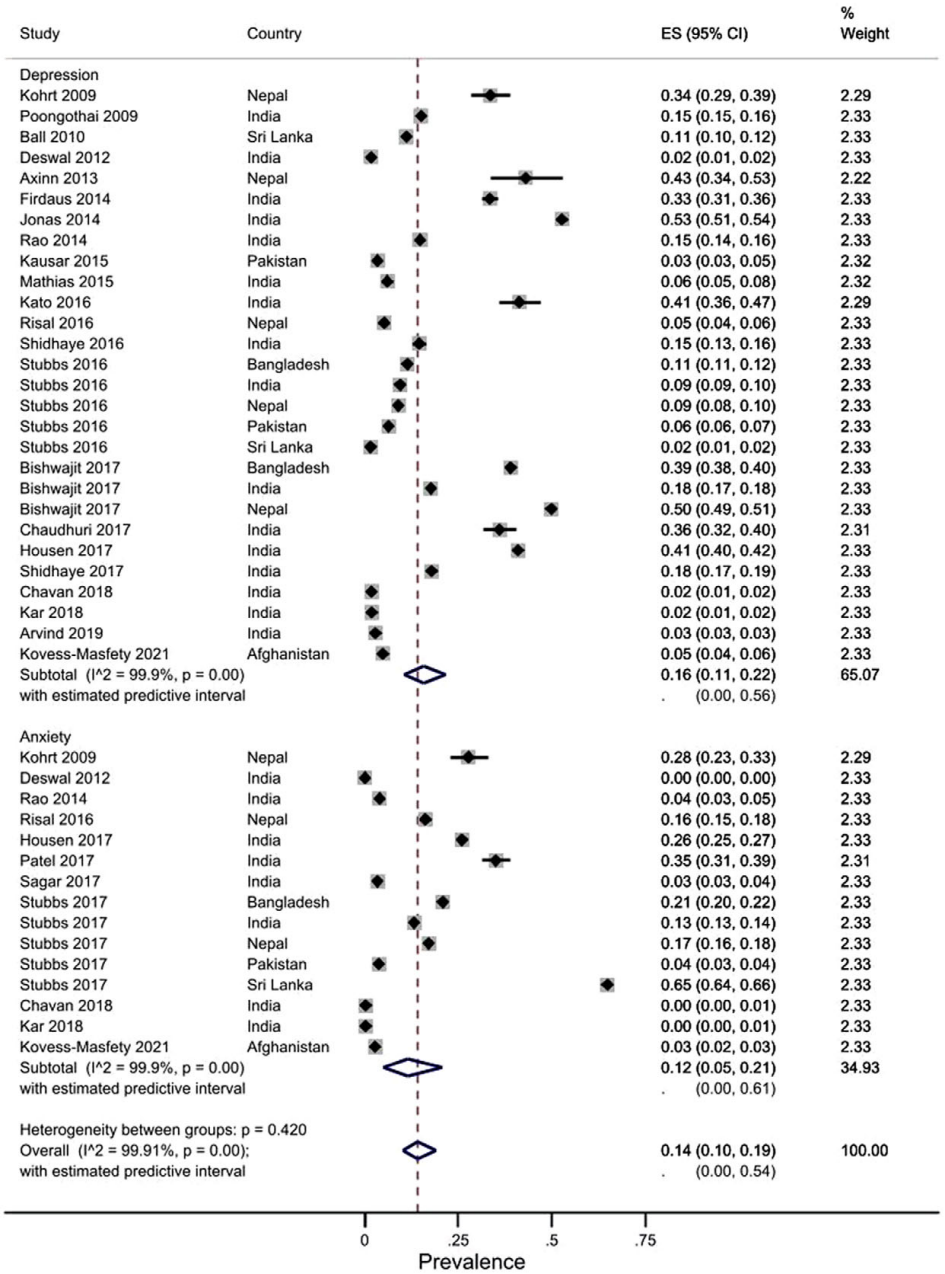
Forest plot of primary studies on the prevalence of depression and anxiety in South Asia.

We identified 25 primary studies reporting the prevalence of CMDs in the general adult population (Table 2): 16 from India, three from Nepal, one each from Pakistan, Sri Lanka, and Afghanistan, and three large, population-based studies that covered multiple countries in SA. Study quality overall was high. Meta-analyses found a pooled prevalence of 16.0% (95% CI = 11.0–22.0, *I* ^2^ = 99.9%) for depression, 12.0% (5.0–21.0, *I* ^2^ = 99.9%) for anxiety, and 14.0% (10.0–19.0, *I* ^2^ = 99.9%) for depression and anxiety combined (Figure 3).

**Table 2.** Summary characteristics of primary studies included in meta-analyses

Study	Country	Setting	Study design	Sample size	Mental disorder(s) and assessment tools used	Quality score
Kohrt et al. (2009)	Nepal	Mixed	Cross-sectional for prevalence	307	Depression – Beck Depression Inventory (BDI); Anxiety – Beck Anxiety Inventory (BAI)	7
Poongothai et al. (2009)	India	Urban	Cross-sectional	25,455	Depression – Modified Patient Health Questionnaire (PHQ12)	9
Ball et al. (2010)	Sri Lanka	Mixed	Cross-sectional	5,973	Depression – Composite International Diagnostic Interview (CIDI)	9
Deswal and Pawar (2012)	India	Urban	Cross-sectional	3,023	Depression, Anxiety – CIDI	9
Axinn et al. (2013)	Nepal	Rural	Cross-sectional	400	Depression – CIDI	7
Firdaus and Ahmad (2014)	India	Urban	Cross-sectional	1,326 in 2003; 1,965 in 2013	Depression – Centre for Epidemiologic Studies Depression Scale (CES-D)	9
Jonas et al. (2014)	India	Rural	Cross-sectional	4,711	Depression – CES-D	8
Rao et al. (2014)	India	Rural	Cross-sectional	3,033	Depression, Anxiety – Mini international neuropsychiatric interview (MINI)	9
Kausar et al. (2015)	Pakistan	Urban	Cross-sectional	1,110	Depression – DSM-based questionnaire	7
Mathias et al. (2015)	India	Mixed	Cross-sectional	960	Depression – PHQ9	9
Kato (2016)	India	NR	Cross-sectional	300	Depression – PHQ9 and CES-D	6
Risal et al. (2016)	Nepal	Mixed	Cross-sectional	2,100	Depression, Anxiety – Hospital Anxiety and Depression Scale (HADS)	9
Shidhaye et al. (2016)	India	Rural	Cross-sectional	1,456	Depression – PHQ9	9
Stubbs et al. (2016)	Bangladesh, India, Nepal, Pakistan, Sri Lanka	Mixed	Cross-sectional	178,867	Depression – Based on DSM	8
Bishwajit et al. (2017)	Bangladesh, India, Nepal	Mixed	Cross-sectional	14,133	Depression – Self-reported	4
Chaudhuri et al. (2017)	India	Mixed	Cross-sectional	469	Depression – BDI	9
Housen et al. (2017)	India	Mixed	Cross-sectional	5,428	Depression, Anxiety – Hopkins Symptom Checklist (HSCL-25)	9
Patel et al. (2017)	India	Urban	Cross-sectional	605	Anxiety – State-Trait Anxiety Inventory (STAI) scale	6
Sagar et al. (2017)	India	Mixed	Cross-sectional	24,371	Anxiety – CIDI	9
Shidhaye et al. (2017)	India	Mixed	Cross-sectional	3,220	Depression – PHQ9	9
Stubbs et al. (2017)	Bangladesh, India, Nepal, Pakistan, Sri Lanka	Mixed	Cross-sectional	237,964	Anxiety – Self-reported	6
Kar et al. (2018)	India	Mixed	Cross-sectional	3,508	Depression, Anxiety – MINI, version 6.0.0	9
Chavan et al. (2018)	India	Mixed	Cross-sectional	2,895	Depression, Anxiety – MINI, version 6.0.0	9
Arvind (2019)	India	Mixed	Cross-sectional	34,802	Depression – MINI, version 6.0.0	9
Kovess-Masfety et al. (2021)	Afghanistan	Mixed	Cross-sectional	4,433	Depression, Anxiety – CIDI	8

The pooled prevalence (95% CI) of depression varied notably by country, from 5.0% (4.0–6.0) in Afghanistan, 5.0% (5.0–6.0) in Sri Lanka and 6.0% (5.0–6.0) in Pakistan to 16.0% (10.0–24.0) in India, 25.0% (6.0–52.0) in Nepal, and 25.0% (24.0–25.0) in Bangladesh. Similarly, the pooled prevalence of anxiety varied between 3.0% (2.0–3.0) in Afghanistan, 4.0% (3.0–4.0) in Pakistan and 6.0% (2.0–14.0) in India, to 19.0% (16.0–23.0) in Nepal, 21.0% (20.0–22.0) in Bangladesh, and 65.0% (64.0–66.0) in Sri Lanka. The pooled values for both conditions also varied markedly according to whether (and which) diagnostic or screening tools were used to ascertain the presence of depression and/or anxiety. For depression, the pooled prevalence from estimates based on diagnostic tools (e.g., Composite International Diagnostic Interview (CIDI) and Mini International Neuro-psychiatric Interview (MINI)) was 5.0% (3.0–6.0), whereas it was 27.0% (13.0–44.0) based on screening measures. Similarly, the pooled prevalence for anxiety from estimates based on diagnostic tools was 1.0% (0.0–3.0), whereas it was 26.0% (19.0–34.0) based on screening measures. Funnel plot asymmetry was observed and Egger’s test for meta-analysis of depression was statistically significant indicating publication bias. Forest plots for subgroup analyses and funnel plots can be found in Supplementary Appendix 9.

## Discussion

This umbrella review has identified many reviews covering a range of mental disorders in SA, with the majority focusing on the prevalence of CMDs among different population groups. Our findings suggest a high prevalence of these conditions in the region, with greater burden among specific population groups, including perinatal women, older people, people with chronic physical illnesses, refugees, and other vulnerable groups. More than 20 reviews were identified on the prevalence of CMDs during COVID-19 and suggest a high burden of mental disorders among healthcare workers, teachers, and students in SA during the pandemic. In common with Hossain et al. (2020) we found that most studies were from India, while evidence from Afghanistan, Bhutan, and Maldives was particularly limited. The advantages and novelty of this review are in providing a more complete and updated picture of the prevalence of mental disorders in the region. But despite the broader inclusion criteria and the updated searches, we found no reviews with pooled estimates of prevalence for many conditions, including severe mental disorders such as schizophrenia and psychotic disorders, behavioural syndromes, personality disorders, or intellectual disabilities. Reviews without meta-analyses for these conditions were also limited. Further, most reviews scored ‘low’ or ‘critically low’ on quality assessment, with very few assessed as providing an accurate and comprehensive summary of available studies on the topic.

Our meta-analysis of primary studies provides pooled estimates for the prevalence of depression and anxiety in the general adult population in SA. We had originally planned to use a 2001 cut-off for the primary studies, set to correspond with the World Health Report on Mental Health (World Health Organization, 2001), but revised this to post-2009 studies, to keep in line with the search period followed by one of the reviews from which we harvested primary studies (Naveed et al., 2020). Similarly, whilst our protocol mentioned meta-analyses for all mental disorders, we limited this step to reviews on CMDs, given these conditions were the focus of the bulk of identified reviews. Both the reviews from which we harvested primary studies had also previously reported pooled estimates for these conditions in SA, but one included studies in all adult populations, including higher-risk perinatal women and older people (Naveed et al., 2020), while the other was limited to studies in Afghanistan and Pakistan (Zuberi et al., 2021). The inclusion of populations with greater disease burden in the former likely explained its higher prevalence compared to our estimates for both depression (26.4% vs. 16.0%) and anxiety (25.8% vs. 12.0%). With regard to the latter review, while reported country-specific pooled estimates are comparable to ours for Pakistan, its estimates are considerably higher for Afghanistan for both conditions (33.0% vs. 5.0% for depression and 25.0% vs. 3.0% for anxiety). This difference may be explained by the inclusion of two studies reporting high prevalence, which were excluded in our review on the basis of publication date (Scholte et al., 2004; Mufti et al., 2005). On the other hand, our searches identified results from a recent national survey on depression and anxiety disorders in Afghanistan, which we included in our meta-analyses (Kovess-Masfety et al., 2021), while the addition of the excluded primary studies from Afghanistan and Pakistan (Nisar et al., 2004) does not considerably change the region-specific pooled estimates for depression or anxiety (Supplementary Appendix 9).

In addition to mental disorders, our umbrella review included 23 reviews on suicide and intentional self-harm, including one review with meta-analysis among adults in SA, which reported a 6.4% pooled prevalence of suicidal behaviours (Naveed et al., 2020). Other reviews found adult suicide rates ranging from 0.43 to 331.0 per 100,000 population, which varied greatly across countries in the region, and in some cases are likely to be gross underestimations of actual rates (Jordans et al., 2014). An even higher prevalence of suicidal behaviours was found among specific population groups, including perinatal women (Fuhr et al., 2014; Amiri and Behnezhad, 2021), people with HIV/AIDS (Collins et al., 2006; Das and Leibowitz, 2011), female sex workers (Somrongthong et al., 2019) and tribal populations (Devarapalli et al., 2020). Three reviews on suicidal behaviours among children and adolescents were identified, all from India (Aggarwal and Berk, 2015; Aggarwal et al., 2017; Ganesan et al., 2020). Further, we found three reviews among suicide and self-harm populations, which reported a high prevalence of mental disorders, particularly depressive disorders (Cho et al., 2016; Ahmed et al., 2017; Knipe et al., 2019).

Our searches identified three reports based on the Global Burden of Disease studies, which we excluded on the basis of study design (Baxter et al., 2016; Liu et al., 2020; Sagar et al., 2020), and because analyses were either limited to just India or estimated annual percentage change in the burden of depression across the region, not directly comparable to the results of our analyses. Similarly, three reviews (Reddy and Chandrashekar, 1998; Ganguli, 2000; Arora and Aeri, 2019) included in the Hossain et al. (2020) umbrella review did not meet our eligibility criteria on study design, but those topics were covered in other included reviews. Our review includes all other reviews they included, but by going beyond geographically limited reviews and summarising the evidence from multi-country reviews that included at least one South Asian country, we have identified many more reviews, providing a more complete picture of the evidence regarding the prevalence of mental disorders in the region. Diverse terms were used to describe the reviews that were included (systematic, scoping, narrative, etc.), but we screened for studies that met our criteria to be considered systematic reviews, and thereby ensured consistency in our inclusions (Haddaway et al., 2022). In addition, our meta-analyses of primary studies on depression and anxiety provide important new information on the prevalence of these conditions among the general adult population in the region.

Some key limitations of the research should be acknowledged. First, our approach for identifying primary studies was through harvesting studies from included reviews and forward citation screening, rather than a systematic search and screening of databases. This may have missed studies and introduced a selection bias, but our pre-defined strategy on having a registered protocol likely protected against this. In addition, there are possibilities of publication bias, which our funnel plots suggested were likely. Our meta-analyses also found high heterogeneity, which could be explained to some extent by differences between countries and assessment tool used, demonstrated by subgroup analyses. The finding that studies using screening tools report higher prevalence than those using diagnostic interviews has been previously reported, which may have overestimated the prevalence of mental disorders (Zuberi et al., 2021). In the methodological literature on clinical trials, developing and adopting ‘core outcome sets’ has been advocated to address the heterogeneity that precludes meaningful synthesis of evidence across studies. Core outcomes sets mandate the inclusion of key outcomes to be measured in all trials of interventions for particular conditions and may also define the tools to be used to measure them (Chiarotto et al., 2017). A similar agreed set of defined measures for observational studies of various mental ill health conditions may be a way forward for better synthesis.

Next, although the majority of primary studies received overall high ratings, few were nationally representative surveys of the general adult population. Nonetheless, there were primary studies from most countries in the region, apart from Bhutan and Maldives. In contrast to the quality of primary studies in our meta-analyses, our narrative synthesis is largely based on reviews that scored ‘low’ or ‘critically low’. We therefore limited our presentation of prevalence estimates solely to the meta-analytical reviews, while the overall narrative summary provides a broader mapping of identified evidence from all reviews by type of review and mental disorder. Finally, there is the possibility that our umbrella review may have missed some relevant reviews on mental disorders in SA, but we searched a large number of (including region-specific) databases and reviewed the literature as comprehensively as possible.

Overall, the findings of our research show a high burden of mental disorders among the general-adult population in SA, with even higher prevalence among specific population subgroups. These findings are also supported by reviews published since our searches were carried out (Manna et al., 2022; Palfreyman and Gazeley, 2022; Al-Mamun et al., 2023; Javan Biparva et al., 2023). Our results highlight an urgent need for countries in SA to formulate and implement both clinical and policy measures for the prevention and early treatment of mental disorders and intentional self-harm. The mapping of evidence according to the type of review and mental disorder (Supplementary Appendix 8) shows that population-level prevalence estimates are generally lacking beyond CMDs, including for schizophrenia and psychotic disorders, behavioural syndromes, personality disorders, and intellectual disabilities. These identified gaps are supported by other recent reviews (Russell et al., 2022; Bastien et al., 2023), and should be a focus of future research, along with the strengthening of epidemiological surveillance systems to better capture morbidity, mortality, and economic burden of all mental disorders and intentional self-harm in the region.

## Supporting information

Vidyasagaran et al. supplementary material Vidyasagaran et al. supplementary material

## Data Availability

The details of data searches and extractions from the included studies are provided in the Supplementary Material. The review protocol, including the analysis plan, can be accessed freely from the PROSPERO database, using the registration number mentioned. We do not have any additional data to share.

## Supplementary material

The supplementary material for this article can be found at https://doi.org/10.1017/gmh.2023.72.
