# Supplementary material for: Prevalence of mental disorders in South Asia: A systematic review of reviews
Source: Glob Ment Health (Camb). 2023 Nov 13;10:e78. doi: 10.1017/gmh.2023.72 (PMC10755414; doi:10.1017/gmh.2023.72)

**Prevalence review – Supplementary material**

# Appendix 1 – PRISMA checklist

| **Section and Topic** | **Item #** | **Checklist item** | **Location where item is reported** |
| --- | --- | --- | --- |
| **TITLE** | | |  |
| Title | 1 | Identify the report as a systematic review. | Pg. 1 |
| **ABSTRACT** | | |  |
| Abstract | 2 | See the PRISMA 2020 for Abstracts checklist. | Pg. 3 |
| **INTRODUCTION** | | |  |
| Rationale | 3 | Describe the rationale for the review in the context of existing knowledge. | Pg. 4 |
| Objectives | 4 | Provide an explicit statement of the objective(s) or question(s) the review addresses. | Pg. 4 |
| **METHODS** | | |  |
| Eligibility criteria | 5 | Specify the inclusion and exclusion criteria for the review and how studies were grouped for the syntheses. | Pg. 5 (also appendix 4) |
| Information sources | 6 | Specify all databases, registers, websites, organisations, reference lists and other sources searched or consulted to identify studies. Specify the date when each source was last searched or consulted. | Pg. 4 (also appendix 2) |
| Search strategy | 7 | Present the full search strategies for all databases, registers, and websites, including any filters and limits used. | Appendix 3 |
| Selection process | 8 | Specify the methods used to decide whether a study met the inclusion criteria of the review, including how many reviewers screened each record and each report retrieved, whether they worked independently, and if applicable, details of automation tools used in the process. | Pg. 5 |
| Data collection process | 9 | Specify the methods used to collect data from reports, including how many reviewers collected data from each report, whether they worked independently, any processes for obtaining or confirming data from study investigators, and if applicable, details of automation tools used in the process. | Pg. 5 |
| Data items | 10a | List and define all outcomes for which data were sought. Specify whether all results that were compatible with each outcome domain in each study were sought (e.g., for all measures, time points, analyses), and if not, the methods used to decide which results to collect. | Pg. 5 |
|  | 10b | List and define all other variables for which data were sought (e.g., participant and intervention characteristics, funding sources). Describe any assumptions made about any missing or unclear information. | Pg. 5 |
| Study risk of bias assessment | 11 | Specify the methods used to assess risk of bias in the included studies, including details of the tool(s) used, how many reviewers assessed each study and whether they worked independently, and if applicable, details of automation tools used in the process. | Pg. 5 |
| Effect measures | 12 | Specify for each outcome the effect measure(s) (e.g., risk ratio, mean difference) used in the synthesis or presentation of results. | Pg. 5 |
| Synthesis methods | 13a | Describe the processes used to decide which studies were eligible for each synthesis (e.g., tabulating the study intervention characteristics and comparing against the planned groups for each synthesis (item #5)). | Pg. 5 and 6 |
|  | 13b | Describe any methods required to prepare the data for presentation or synthesis, such as handling of missing summary statistics, or data conversions. | Pg. 5 and 6 |
|  | 13c | Describe any methods used to tabulate or visually display results of individual studies and syntheses. | Pg. 5 and 6 |
|  | 13d | Describe any methods used to synthesize results and provide a rationale for the choice(s). If meta-analysis was performed, describe the model(s), method(s) to identify the presence and extent of statistical heterogeneity, and software package(s) used. | Pg. 5 and 6 |
|  | 13e | Describe any methods used to explore possible causes of heterogeneity among study results (e.g. subgroup analysis, meta-regression). | Pg. 5 and 6 |
|  | 13f | Describe any sensitivity analyses conducted to assess robustness of the synthesized results. | NA |
| Reporting bias assessment | 14 | Describe any methods used to assess risk of bias due to missing results in a synthesis (arising from reporting biases). | Pg. 5 and 6 |
| Certainty assessment | 15 | Describe any methods used to assess certainty (or confidence) in the body of evidence for an outcome. | NA |
| **RESULTS** | | |  |
| Study selection | 16a | Describe the results of the search and selection process, from the number of records identified in the search to the number of studies included in the review, ideally using a flow diagram. | Pg. 6 |
|  | 16b | Cite studies that might appear to meet the inclusion criteria, but which were excluded, and explain why they were excluded. | Pg. 6 (also appendix 5) |
| Study characteristics | 17 | Cite each included study and present its characteristics. | Pg. 8 and Table 1 (also appendix 5) |
| Risk of bias in studies | 18 | Present assessments of risk of bias for each included study. | Pg. 8 and Table 1 (also appendix 5) |
| Results of individual studies | 19 | For all outcomes, present, for each study: (a) summary statistics for each group (where appropriate) and (b) an effect estimate and its precision (e.g. confidence/credible interval), ideally using structured tables or plots. | Table 1; also Pgs. 17-20 |
| Results of syntheses | 20a | For each synthesis, briefly summarise the characteristics and risk of bias among contributing studies. | Pgs. 8 and 22 |
|  | 20b | Present results of all statistical syntheses conducted. If meta-analysis was done, present for each the summary estimate and its precision (e.g. confidence/credible interval) and measures of statistical heterogeneity. If comparing groups, describe the direction of the effect. | Pgs. 22 and 23 |
|  | 20c | Present results of all investigations of possible causes of heterogeneity among study results. | Pg. 22 (also appendix 9) |
|  | 20d | Present results of all sensitivity analyses conducted to assess the robustness of the synthesized results. | NA |
| Reporting biases | 21 | Present assessments of risk of bias due to missing results (arising from reporting biases) for each synthesis assessed. | Pgs. 22 and 23 (also appendix 9) |
| Certainty of evidence | 22 | Present assessments of certainty (or confidence) in the body of evidence for each outcome assessed. | NA |
| **DISCUSSION** | | |  |
| Discussion | 23a | Provide a general interpretation of the results in the context of other evidence. | Pgs. 23 and 24 |
|  | 23b | Discuss any limitations of the evidence included in the review. | Pgs. 24 and 25 |
|  | 23c | Discuss any limitations of the review processes used. | Pg. 25 |
|  | 23d | Discuss implications of the results for practice, policy, and future research. | Pg. 25 |
| **OTHER INFORMATION** | | |  |
| Registration and protocol | 24a | Provide registration information for the review, including register name and registration number, or state that the review was not registered. | Pg. 4 |
|  | 24b | Indicate where the review protocol can be accessed, or state that a protocol was not prepared. | PROSPERO |
|  | 24c | Describe and explain any amendments to information provided at registration or in the protocol. | Pg. 24 |
| Support | 25 | Describe sources of financial or non-financial support for the review, and the role of the funders or sponsors in the review. | Abstract, Pgs. 6 and 26 |
| Competing interests | 26 | Declare any competing interests of review authors. | Pg. 26 |
| Availability of data, code and other materials | 27 | Report which of the following are publicly available and where they can be found: template data collection forms; data extracted from included studies; data used for all analyses; analytic code; any other materials used in the review. | Extracted data – Tables and appendices |

# Appendix 2 – Databases and repositories searched

| **Content type** | **Database or repository name** | **Database or repository platform or URL** |
| --- | --- | --- |
| Published literature | Embase Classic+Embase  <1947 to 2021 September 27> | Ovid |
|  | Epistemonikos | https://www.epistemonikos.org/ |
|  | Global Health  <1910 to 2021 Week 38> | Ovid |
|  | MEDLINE(R) ALL  <1946 to September 27, 2021> | Ovid |
|  | PakMediNet | https://www.pakmedinet.com/ |
|  | APA PsycInfo  <1806 to September Week 3 2021> | Ovid |
| Unpublished literature | ELDIS | https://www.eldis.org/ |
|  | WHO IRIS Institutional Repository for Information Sharing | https://apps.who.int/iris/ |
|  | World Bank Open Knowledge Repository | https://openknowledge.worldbank.org/ |

# Appendix 3 – Search strategies

Literature search for systematic review of Prevalence of Mental Disorders in South Asia, 29-09-2021

## Embase Classic+Embase (Ovid) <1947 to 2021 September 27>

Search date: 29/09/2021

Records found: 338

1 ((Indian or Indians) not ("west indian*" or "american indian*")).ti,ab,in. (184895)

2 india.ti,ab,in. (969890)

3 bangladesh*.ti,ab,in. (36924)

4 pakistan*.ti,ab,in. (104046)

5 "south asia*".ti,ab,in. (15316)

6 afghanistan*.ti,ab,in. (8385)

7 Bhutan*.ti,ab,in. (3330)

8 nepal*.ti,ab,in. (23533)

9 "sri lanka*".ti,ab,in. (17639)

10 maldives.ti,ab,in. (544)

11 exp south asia/ or Maldives/ (245328)

12 or/1-11 [S Asia] (1220083)

13 exp mental disease/ not exp motor dysfunction/ (2254032)

14 automutilation/ or self poisoning/ (22731)

15 suicide/ or suicide attempt/ (91971)

16 exp speech disorder/ (106416)

17 exp mental deficiency/ (161462)

18 or/13-17 (2350121)

19 prevalence/ (810413)

20 incidence/ (473154)

21 epidemiology/ (242648)

22 *cross-sectional study/ (12620)

23 *cohort analysis/ (41011)

24 *follow up/ (52739)

25 exp *case control study/ (9840)

26 exp *longitudinal study/ (9146)

27 *prospective study/ (33235)

28 *retrospective study/ (33321)

29 or/19-28 [Prevalence or longitudinal studies EMTREE search] (1592349)

30 18 and 29 (174105)

31 exp drug dependence/ep (15149)

32 30 or 31 [Prevalence or longitudinal studies of mental illness EMTREE search] (184803)

33 ((prevalen* or incidence* or "risk factor*" or epidemiolog*) adj4 (mental or mentally or psychiatr* or psycho* or depressi* or depressed or MDD or anxi* or phobia or phobic or agoraphobi* or dysthymi* or ADNOS)).ti,kw. (19642)

34 ((prevalen* or incidence* or "risk factor*" or epidemiolog*) adj4 (schizo* or hebephrenic* or oligophreni* or akathisi* or acathisi* or neuroleptic-induc*)).ti,kw. (1953)

35 ((prevalen* or incidence* or "risk factor*" or epidemiolog*) adj4 (tardiv* adj dyskine*)).ti,kw. (152)

36 ((prevalen* or incidence* or "risk factor*" or epidemiolog*) adj4 (somatoform or somatiz* or somatis* or hysteri* or briquet or multisomat* or multi somat* or MUPs or medically unexplained)).ti,kw. (124)

37 ((prevalen* or incidence* or "risk factor*" or epidemiolog*) adj4 ((dissociative adj3 (disorder* or reaction*)) or dissociation)).ti,kw. (42)

38 ((prevalen* or incidence* or "risk factor*" or epidemiolog*) adj4 (affective* adj (disorder? or disease? or illness* or symptom?))).ti,kw. (108)

39 ((prevalen* or incidence* or "risk factor*" or epidemiolog*) adj4 (PTSD or psychological trauma or psychotrauma* or combat disorder? or war disorder?)).ti,kw. (411)

40 ((prevalen* or incidence* or "risk factor*" or epidemiolog*) adj4 ((post-trauma* or posttrauma*) adj3 (stress* or disorder?))).ti,kw. (581)

41 ((prevalen* or incidence* or "risk factor*" or epidemiolog*) adj4 ((stress or cognitive or cognition or personality or impulse control or mood or paranoid or psychotic or neurologic* or nervous or eating) adj (disorder? or illness* or disease?))).ti,kw. (1526)

42 ((prevalen* or incidence* or "risk factor*" or epidemiolog*) adj4 ((bipolar or behavio?ral or obsessive or compulsive or panic or mood or delusional) adj (disorder? or illness* or disease?))).ti,kw. (561)

43 ((prevalen* or incidence* or "risk factor*" or epidemiolog*) adj4 (trichotillomani* or OCD or obsess*-compulsi* or GAD or stress reaction? or acute stress or neuros#s or neurotic)).ti,kw. (450)

44 ((prevalen* or incidence* or "risk factor*" or epidemiolog*) adj4 (stress syndrome? or distress syndrome? or pain disorder? or dementia or alzheimer*)).ti,kw. (5461)

45 ((prevalen* or incidence* or "risk factor*" or epidemiolog*) adj4 ((substance abuse or "substance use" or drug abuse or "drug use") adj2 disorder?)).ti,kw. (154)

46 ((prevalen* or incidence* or "risk factor*" or epidemiolog*) adj4 (sleep? adj2 (disorder? or syndrome?))).ti,kw. (533)

47 ((prevalen* or incidence* or "risk factor*" or epidemiolog*) adj4 (manic or mania or amnesic syndrome? or severe stress or adjustment disorder? or behavio?ral syndrome? or habit disorder? or hallucinosis or catatonic disorder?)).ti,kw. (111)

48 ((prevalen* or incidence* or "risk factor*" or epidemiolog*) adj4 ((sexual* or gender* or psychosexual*) adj3 (dysfunction* or disorder? or dysphoria))).ti,kw. (499)

49 ((prevalen* or incidence* or "risk factor*" or epidemiolog*) adj4 (transsexual* or transvest* or fetish* or exhibitionis* or voyeuris* or p?edophil* or sadomasochis*)).ti,kw. (44)

50 ((prevalen* or incidence* or "risk factor*" or epidemiolog*) adj4 (autism or autistic or "rett syndrome?" or asperger* or dyspraxia or (developmental adj3 disorder?))).ti,kw. (1059)

51 ((prevalen* or incidence* or "risk factor*" or epidemiolog*) adj4 (aphasia or dysphasia or ((speech or language) adj3 disorder?))).ti,kw. (76)

52 ((prevalen* or incidence* or "risk factor*" or epidemiolog*) adj4 ((intellectual or mental or learning) adj2 (disab* or disorder* or retard* or difficulties))).ti,kw. (1253)

53 ((prevalen* or incidence* or "risk factor*" or epidemiolog*) adj4 ((emotion* or hyperkinetic or conduct or "social function*" or tic) adj2 disorder?)).ti,kw. (74)

54 ((prevalen* or incidence* or "risk factor*" or epidemiolog*) adj4 ((self or themsel* or onesel*) adj2 (aggress* or harm* or cutt* or immolat* or inflict* or injur* or mutilat* or poison* or damag* or destruct*))).ti,kw. (263)

55 ((prevalen* or incidence* or "risk factor*" or epidemiolog*) adj4 (parasuicid* or para-suicid* or suicidality or NSSI or autoaggress* or "auto aggress*" or auto-aggress)).ti,kw. (206)

56 ((prevalen* or incidence* or "risk factor*" or epidemiolog*) adj4 (suicid* adj2 (death or die* or mortality or complete or attempt* or behavio* or intent* or intend* or commit*))).ti,kw. (736)

57 ((prevalen* or incidence* or "risk factor*" or epidemiolog*) adj4 (overdos* adj2 (deliberat* or intentional or intended))).ti,kw. (6)

58 (rate? adj4 ((self or themsel* or onesel*) adj2 (aggress* or harm* or cutt* or immolat* or inflict* or injur* or mutilat* or poison* or damag* or destruct*))).ti,kw. (45)

59 (rate? adj4 (parasuicid* or para-suicid* or suicidality or NSSI or autoaggress* or "auto aggress*" or auto-aggress)).ti,kw. (11)

60 (rate? adj4 (suicid* adj2 (death or die* or mortality or complete or attempt* or behavio* or intent* or intend* or commit*))).ti,kw. (103)

61 (rate? adj4 (overdos* adj2 (deliberat* or intentional or intended))).ti,kw. (0)

62 ((case-control or cohort or follow-up or follow-on or longitudinal or prospective or retrospective or cross-sectional) adj8 (mental or mentally or psychiatr* or psycho* or depressi* or depressed or MDD or anxi* or phobia or phobic or agoraphobi* or dysthymi* or ADNOS)).ti,kw. (20515)

63 ((case-control or cohort or follow-up or follow-on or longitudinal or prospective or retrospective or cross-sectional) adj8 (schizo* or hebephrenic* or oligophreni* or akathisi* or acathisi* or neuroleptic-induc*)).ti,kw. (3415)

64 ((case-control or cohort or follow-up or follow-on or longitudinal or prospective or retrospective or cross-sectional) adj8 (tardiv* adj dyskine*)).ti,kw. (72)

65 ((case-control or cohort or follow-up or follow-on or longitudinal or prospective or retrospective or cross-sectional) adj8 (somatoform or somatiz* or somatis* or hysteri* or briquet or multisomat* or multi somat* or MUPs or medically unexplained)).ti,kw. (111)

66 ((case-control or cohort or follow-up or follow-on or longitudinal or prospective or retrospective or cross-sectional) adj8 ((dissociative adj3 (disorder* or reaction*)) or dissociation)).ti,kw. (124)

67 ((case-control or cohort or follow-up or follow-on or longitudinal or prospective or retrospective or cross-sectional) adj8 (affective* adj (disorder? or disease? or illness* or symptom?))).ti,kw. (177)

68 ((case-control or cohort or follow-up or follow-on or longitudinal or prospective or retrospective or cross-sectional) adj8 (PTSD or psychological trauma or psychotrauma* or combat disorder? or war disorder?)).ti,kw. (309)

69 ((case-control or cohort or follow-up or follow-on or longitudinal or prospective or retrospective or cross-sectional) adj8 ((post-trauma* or posttrauma*) adj3 (stress* or disorder?))).ti,kw. (555)

70 ((case-control or cohort or follow-up or follow-on or longitudinal or prospective or retrospective or cross-sectional) adj8 ((stress or cognitive or cognition or personality or impulse control or mood or paranoid or psychotic or neurologic* or nervous or eating) adj (disorder? or illness* or disease?))).ti,kw. (1612)

71 ((case-control or cohort or follow-up or follow-on or longitudinal or prospective or retrospective or cross-sectional) adj8 ((bipolar or behavio?ral or obsessive or compulsive or panic or mood or delusional) adj (disorder? or illness* or disease?))).ti,kw. (1415)

72 ((case-control or cohort or follow-up or follow-on or longitudinal or prospective or retrospective or cross-sectional) adj8 (trichotillomani* or OCD or obsess*-compulsi* or GAD or stress reaction? or acute stress or neuros#s or neurotic)).ti,kw. (502)

73 ((case-control or cohort or follow-up or follow-on or longitudinal or prospective or retrospective or cross-sectional) adj8 (stress syndrome? or distress syndrome? or pain disorder? or dementia or alzheimer*)).ti,kw. (5277)

74 ((case-control or cohort or follow-up or follow-on or longitudinal or prospective or retrospective or cross-sectional) adj8 (sleep? adj2 (disorder? or syndrome?))).ti,kw. (410)

75 ((case-control or cohort or follow-up or follow-on or longitudinal or prospective or retrospective or cross-sectional) adj8 (sleep? adj2 (disorder? or syndrome?))).ti,kw. (410)

76 ((case-control or cohort or follow-up or follow-on or longitudinal or prospective or retrospective or cross-sectional) adj8 (manic or mania or amnesic syndrome? or severe stress or adjustment disorder? or behavio?ral syndrome? or habit disorder? or hallucinosis or catatonic disorder?)).ti,kw. (236)

77 ((case-control or cohort or follow-up or follow-on or longitudinal or prospective or retrospective or cross-sectional) adj8 ((sexual* or gender* or psychosexual*) adj3 (dysfunction* or disorder? or dysphoria))).ti,kw. (265)

78 ((case-control or cohort or follow-up or follow-on or longitudinal or prospective or retrospective or cross-sectional) adj8 (transsexual* or transvest* or fetish* or exhibitionis* or voyeuris* or p?edophil* or sadomasochis*)).ti,kw. (60)

79 ((case-control or cohort or follow-up or follow-on or longitudinal or prospective or retrospective or cross-sectional) adj8 (autism or autistic or "rett syndrome?" or asperger* or dyspraxia or (developmental adj3 disorder?))).ti,kw. (1206)

80 ((case-control or cohort or follow-up or follow-on or longitudinal or prospective or retrospective or cross-sectional) adj8 (aphasia or dysphasia or ((speech or language) adj3 disorder?))).ti,kw. (175)

81 ((case-control or cohort or follow-up or follow-on or longitudinal or prospective or retrospective or cross-sectional) adj8 ((intellectual or mental or learning) adj2 (disab* or disorder* or retard* or difficulties))).ti,kw. (927)

82 ((case-control or cohort or follow-up or follow-on or longitudinal or prospective or retrospective or cross-sectional) adj8 ((emotion* or hyperkinetic or conduct or "social function*" or tic) adj2 disorder?)).ti,kw. (105)

83 ((case-control or cohort or follow-up or follow-on or longitudinal or prospective or retrospective or cross-sectional) adj8 ((self or themsel* or onesel*) adj2 (aggress* or harm* or cutt* or immolat* or inflict* or injur* or mutilat* or poison* or damag* or destruct*))).ti,kw. (468)

84 ((case-control or cohort or follow-up or follow-on or longitudinal or prospective or retrospective or cross-sectional) adj8 (parasuicid* or para-suicid* or suicidality or NSSI or autoaggress* or "auto aggress*" or auto-aggress)).ti,kw. (154)

85 ((case-control or cohort or follow-up or follow-on or longitudinal or prospective or retrospective or cross-sectional) adj8 (suicid* adj2 (death or die* or mortality or complete or attempt* or behavio* or intent* or intend* or commit*))).ti,kw. (812)

86 ((case-control or cohort or follow-up or follow-on or longitudinal or prospective or retrospective or cross-sectional) adj8 (overdos* adj2 (deliberat* or intentional or intended))).ti,kw. (8)

87 or/33-86 [Prevalence or longitudinal studies of mental illness TEXTWORD search] (66331)

88 32 or 87 [Prevalence of Mental Illness] (229463)

89 12 and 88 (9596)

90 exp meta analysis/ or "systematic review"/ (422956)

91 (Literature review* or (systematic adj2 review*) or (narrative adj2 review*) or (critical adj2 review*) or (evidence adj2 synthesis) or scoping review* or meta-analys* or "meta analysis").ti. (367233)

92 90 or 91 (540927)

93 89 and 92 (338)

## Epistemonikos https://www.epistemonikos.org/

Search date: 29/09/2021

Records found: 237

(advanced_title_en:(mental* OR psychiatri* OR psycholog* OR depress* OR anxiety OR phobia OR schizophreni* OR psychosis OR psychotic OR bipolar OR somatoform OR stress OR mood OR behavioral OR behavioural OR neuroses OR personality OR dementia* OR alzheimer* OR suicid* OR "self harm" OR "intellectual disabilit*" OR "speech disorder*" OR intellectual OR speech) OR advanced_abstract_en:(mental* OR psychiatri* OR psycholog* OR depress* OR anxiety OR phobia OR schizophreni* OR psychosis OR psychotic OR bipolar OR somatoform OR stress OR mood OR behavioral OR behavioural OR neuroses OR personality OR dementia* OR alzheimer* OR suicid* OR "self harm" OR "intellectual disabilit*" OR "speech disorder*" OR intellectual OR speech)) AND (advanced_title_en:(prevalen* OR incidence* OR "risk factor*" OR epidemiolog* OR rate OR rates) OR advanced_abstract_en:(prevalen* OR incidence* OR "risk factor*" OR epidemiolog* OR rate OR rates)) AND (advanced_title_en:(afghanistan* OR bangladesh* OR Bhutan* OR india* OR maldives OR nepal* OR pakistan* OR "south asia*" OR "sri lanka*") OR advanced_abstract_en:(afghanistan* OR bangladesh* OR Bhutan* OR india* OR maldives OR nepal* OR pakistan* OR "south asia*" OR "sri lanka*")) [Filters: classification=systematic-review, protocol=no]

## Global Health (Ovid) <1910 to 2021 Week 38>

Search date: 29/09/2021

Records found: 55

1 ((Indian or Indians) not ("west indian*" or "american indian*")).ti,ab,id,gl. (38704)

2 india.ti,ab,id,gl. (143123)

3 afghanistan*.ti,ab,id,gl. (2703)

4 bangladesh*.ti,ab,id,gl. (14790)

5 Bhutan*.ti,ab,id,gl. (759)

6 nepal*.ti,ab,id,gl. (8739)

7 pakistan*.ti,ab,id,gl. (21555)

8 "south asia*".ti,ab,id,gl. (5131)

9 "sri lanka*".ti,ab,id,gl. (8024)

10 maldives.ti,ab,id,gl. (282)

11 exp south asia/ or afghanistan/ or maldives/ (181344)

12 or/1-11 [S Asia] (208036)

13 exp mental disorders/ not exp movement disorders/ (86180)

14 suicide/ (8763)

15 people with speech impairment/ (77)

16 mental retardation/ (1821)

17 exp substance abuse/ (29467)

18 or/13-17 (114622)

19 risk factors/ (266646)

20 disease incidence/ (60284)

21 disease prevalence/ (181688)

22 longitudinal studies/ (9966)

23 cohort studies/ (67884)

24 retrospective studies/ (20387)

25 follow up/ (26257)

26 epidemiological surveys/ (15197)

27 rates/ (261)

28 or/19-27 (510524)

29 13 and 28 [Prevalence or longitudinal studies of mental illness subject heading search] (27925)

30 ((prevalen* or incidence* or "risk factor*" or epidemiolog*) adj4 (mental or mentally or psychiatr* or psycho* or depressi* or depressed or MDD or anxi* or phobia or phobic or agoraphobi* or dysthymi* or ADNOS)).ti,id. (2409)

31 ((prevalen* or incidence* or "risk factor*" or epidemiolog*) adj4 (schizo* or hebephrenic* or oligophreni* or akathisi* or acathisi* or neuroleptic-induc*)).ti,id. (107)

32 ((prevalen* or incidence* or "risk factor*" or epidemiolog*) adj4 (tardiv* adj dyskine*)).ti,id. (3)

33 ((prevalen* or incidence* or "risk factor*" or epidemiolog*) adj4 (somatoform or somatiz* or somatis* or hysteri* or briquet or multisomat* or multi somat* or MUPs or medically unexplained)).ti,id. (13)

34 ((prevalen* or incidence* or "risk factor*" or epidemiolog*) adj4 ((dissociative adj3 (disorder* or reaction*)) or dissociation)).ti,id. (2)

35 ((prevalen* or incidence* or "risk factor*" or epidemiolog*) adj4 (affective* adj (disorder? or disease? or illness* or symptom?))).ti,id. (10)

36 ((prevalen* or incidence* or "risk factor*" or epidemiolog*) adj4 (PTSD or psychological trauma or psychotrauma* or combat disorder? or war disorder?)).ti,id. (25)

37 ((prevalen* or incidence* or "risk factor*" or epidemiolog*) adj4 ((post-trauma* or posttrauma*) adj3 (stress* or disorder?))).ti,id. (102)

38 ((prevalen* or incidence* or "risk factor*" or epidemiolog*) adj4 ((stress or cognitive or cognition or personality or impulse control or mood or paranoid or psychotic or neurologic* or nervous or eating) adj (disorder? or illness* or disease?))).ti,id. (351)

39 ((prevalen* or incidence* or "risk factor*" or epidemiolog*) adj4 ((bipolar or behavio?ral or obsessive or compulsive or panic or mood or delusional) adj (disorder? or illness* or disease?))).ti,id. (74)

40 ((prevalen* or incidence* or "risk factor*" or epidemiolog*) adj4 (trichotillomani* or OCD or obsess*-compulsi* or GAD or stress reaction? or acute stress or neuros#s or neurotic)).ti,id. (35)

41 ((prevalen* or incidence* or "risk factor*" or epidemiolog*) adj4 (stress syndrome? or distress syndrome? or pain disorder? or dementia or alzheimer*)).ti,id. (641)

42 ((prevalen* or incidence* or "risk factor*" or epidemiolog*) adj4 ((substance abuse or "substance use" or drug abuse or "drug use") adj2 disorder?)).ti,id. (38)

43 ((prevalen* or incidence* or "risk factor*" or epidemiolog*) adj4 (sleep? adj2 (disorder? or syndrome?))).ti,id. (66)

44 ((prevalen* or incidence* or "risk factor*" or epidemiolog*) adj4 (manic or mania or amnesic syndrome? or severe stress or adjustment disorder? or behavio?ral syndrome? or habit disorder? or hallucinosis or catatonic disorder?)).ti,id. (6)

45 ((prevalen* or incidence* or "risk factor*" or epidemiolog*) adj4 ((sexual* or gender* or psychosexual*) adj3 (dysfunction* or disorder? or dysphoria))).ti,id. (63)

46 ((prevalen* or incidence* or "risk factor*" or epidemiolog*) adj4 (transsexual* or transvest* or fetish* or exhibitionis* or voyeuris* or p?edophil* or sadomasochis*)).ti,id. (4)

47 ((prevalen* or incidence* or "risk factor*" or epidemiolog*) adj4 (autism or autistic or "rett syndrome?" or asperger* or dyspraxia or (developmental adj3 disorder?))).ti,id. (175)

48 ((prevalen* or incidence* or "risk factor*" or epidemiolog*) adj4 (aphasia or dysphasia or ((speech or language) adj3 disorder?))).ti,id. (5)

49 ((prevalen* or incidence* or "risk factor*" or epidemiolog*) adj4 ((intellectual or mental or learning) adj2 (disab* or disorder* or retard* or difficulties))).ti,id. (247)

50 ((prevalen* or incidence* or "risk factor*" or epidemiolog*) adj4 ((emotion* or hyperkinetic or conduct or "social function*" or tic) adj2 disorder?)).ti,id. (13)

51 ((prevalen* or incidence* or "risk factor*" or epidemiolog*) adj4 ((self or themsel* or onesel*) adj2 (aggress* or harm* or cutt* or immolat* or inflict* or injur* or mutilat* or poison* or damag* or destruct*))).ti,id. (61)

52 ((prevalen* or incidence* or "risk factor*" or epidemiolog*) adj4 (parasuicid* or para-suicid* or suicidality or NSSI or autoaggress* or "auto aggress*" or auto-aggress)).ti,id. (17)

53 ((prevalen* or incidence* or "risk factor*" or epidemiolog*) adj4 (suicid* adj2 (death or die* or mortality or complete or attempt* or behavio* or intent* or intend* or commit*))).ti,id. (135)

54 ((prevalen* or incidence* or "risk factor*" or epidemiolog*) adj4 (overdose* adj2 (deliberat* or intentional or intended))).ti,id. (2)

55 (rate? adj4 ((self or themsel* or onesel*) adj2 (aggress* or harm* or cutt* or immolat* or inflict* or injur* or mutilat* or poison* or damag* or destruct*))).ti,id. (8)

56 (rate? adj4 (parasuicid* or para-suicid* or suicidality or NSSI or autoaggress* or "auto aggress*" or auto-aggress)).ti,id. (0)

57 (rate? adj4 (suicid* adj2 (death or die* or mortality or complete or attempt* or behavio* or intent* or intend* or commit*))).ti,id. (28)

58 (rate? adj4 (overdose* adj2 (deliberat* or intentional or intended))).ti,id. (0)

59 ((case-control or cohort or follow-up or follow-on or longitudinal or prospective or retrospective or cross-sectional) adj8 (mental or mentally or psychiatr* or psycho* or depressi* or depressed or MDD or anxi* or phobia or phobic or agoraphobi* or dysthymi* or ADNOS)).ti,id. (2432)

60 ((case-control or cohort or follow-up or follow-on or longitudinal or prospective or retrospective or cross-sectional) adj8 (schizo* or hebephrenic* or oligophreni* or akathisi* or acathisi* or neuroleptic-induc*)).ti,id. (168)

61 ((case-control or cohort or follow-up or follow-on or longitudinal or prospective or retrospective or cross-sectional) adj8 (tardiv* adj dyskine*)).ti,id. (1)

62 ((case-control or cohort or follow-up or follow-on or longitudinal or prospective or retrospective or cross-sectional) adj8 (somatoform or somatiz* or somatis* or hysteri* or briquet or multisomat* or multi somat* or MUPs or medically unexplained)).ti,id. (4)

63 ((case-control or cohort or follow-up or follow-on or longitudinal or prospective or retrospective or cross-sectional) adj8 ((dissociative adj3 (disorder* or reaction*)) or dissociation)).ti,id. (1)

64 ((case-control or cohort or follow-up or follow-on or longitudinal or prospective or retrospective or cross-sectional) adj8 (affective* adj (disorder? or disease? or illness* or symptom?))).ti,id. (8)

65 ((case-control or cohort or follow-up or follow-on or longitudinal or prospective or retrospective or cross-sectional) adj8 (PTSD or psychological trauma or psychotrauma* or combat disorder? or war disorder?)).ti,id. (11)

66 ((case-control or cohort or follow-up or follow-on or longitudinal or prospective or retrospective or cross-sectional) adj8 ((post-trauma* or posttrauma*) adj3 (stress* or disorder?))).ti,id. (63)

67 ((case-control or cohort or follow-up or follow-on or longitudinal or prospective or retrospective or cross-sectional) adj8 ((stress or cognitive or cognition or personality or impulse control or mood or paranoid or psychotic or neurologic* or nervous or eating) adj (disorder? or illness* or disease?))).ti,id. (279)

68 ((case-control or cohort or follow-up or follow-on or longitudinal or prospective or retrospective or cross-sectional) adj8 ((bipolar or behavio?ral or obsessive or compulsive or panic or mood or delusional) adj (disorder? or illness* or disease?))).ti,id. (71)

69 ((case-control or cohort or follow-up or follow-on or longitudinal or prospective or retrospective or cross-sectional) adj8 (trichotillomani* or OCD or obsess*-compulsi* or GAD or stress reaction? or acute stress or neuros#s or neurotic)).ti,id. (13)

70 ((case-control or cohort or follow-up or follow-on or longitudinal or prospective or retrospective or cross-sectional) adj8 (stress syndrome? or distress syndrome? or pain disorder? or dementia or alzheimer?)).ti,id. (544)

71 ((case-control or cohort or follow-up or follow-on or longitudinal or prospective or retrospective or cross-sectional) adj8 ((substance abuse or "substance use" or drug abuse or "drug use") adj2 disorder?)).ti,id. (42)

72 ((case-control or cohort or follow-up or follow-on or longitudinal or prospective or retrospective or cross-sectional) adj8 (sleep? adj2 (disorder? or syndrome?))).ti,id. (23)

73 ((case-control or cohort or follow-up or follow-on or longitudinal or prospective or retrospective or cross-sectional) adj8 (manic or mania or amnesic syndrome? or severe stress or adjustment disorder? or behavio?ral syndrome? or habit disorder? or hallucinosis or catatonic disorder?)).ti,id. (7)

74 ((case-control or cohort or follow-up or follow-on or longitudinal or prospective or retrospective or cross-sectional) adj8 ((sexual* or gender* or psychosexual*) adj3 (dysfunction* or disorder? or dysphoria))).ti,id. (11)

75 ((case-control or cohort or follow-up or follow-on or longitudinal or prospective or retrospective or cross-sectional) adj8 (transsexual* or transvest* or fetish* or exhibitionis* or voyeuris* or p?edophil* or sadomasochis*)).ti,id. (0)

76 ((case-control or cohort or follow-up or follow-on or longitudinal or prospective or retrospective or cross-sectional) adj8 (autism or autistic or "rett syndrome?" or asperger* or dyspraxia or (developmental adj3 disorder?))).ti,id. (137)

77 ((case-control or cohort or follow-up or follow-on or longitudinal or prospective or retrospective or cross-sectional) adj8 (aphasia or dysphasia or ((speech or language) adj3 disorder?))).ti,id. (3)

78 ((case-control or cohort or follow-up or follow-on or longitudinal or prospective or retrospective or cross-sectional) adj8 ((intellectual or mental or learning) adj2 (disab* or disorder* or retard* or difficulties))).ti,id. (153)

79 ((case-control or cohort or follow-up or follow-on or longitudinal or prospective or retrospective or cross-sectional) adj8 ((emotion* or hyperkinetic or conduct or "social function*" or tic) adj2 disorder?)).ti,id. (8)

80 ((case-control or cohort or follow-up or follow-on or longitudinal or prospective or retrospective or cross-sectional) adj8 ((self or themsel* or onesel*) adj2 (aggress* or harm* or cutt* or immolat* or inflict* or injur* or mutilat* or poison* or damag* or destruct*))).ti,id. (82)

81 ((case-control or cohort or follow-up or follow-on or longitudinal or prospective or retrospective or cross-sectional) adj8 (parasuicid* or para-suicid* or suicidality or NSSI or autoaggress* or "auto aggress*" or auto-aggress)).ti,id. (17)

82 ((case-control or cohort or follow-up or follow-on or longitudinal or prospective or retrospective or cross-sectional) adj8 (suicid* adj2 (death or die* or mortality or complete or attempt* or behavio* or intent* or intend* or commit*))).ti,id. (168)

83 ((case-control or cohort or follow-up or follow-on or longitudinal or prospective or retrospective or cross-sectional) adj8 (overdos* adj2 (deliberat* or intentional or intended))).ti,id. (1)

84 or/30-83 [Prevalence or longitudinal studies of mental illness TEXTWORD search] (7890)

85 29 or 84 (31120)

86 12 and 85 (1465)

87 systematic reviews/ or meta-analysis/ (57092)

88 (Literature review* or (systematic adj2 review*) or (narrative adj2 review*) or (critical adj2 review*) or (evidence adj2 synthesis) or scoping review* or meta-analys* or "meta analysis").ti. (56542)

89 87 or 88 (69030)

90 86 and 89 (55)

## IMSEAR Index Medicus for the South East Asia Region (WHO Global Health Index Medicus)

Search date: 29/09/2021

Records found: 70

(ti:(intellectual OR speech OR mental OR psychiatric OR psychological OR depression OR anxiety OR phobia OR schizophrenia OR psychosis OR psychotic OR psychoses OR bipolar OR somatoform OR stress OR mood OR behavioral OR neuroses OR behavioural OR personality OR dementia OR alzheimer* OR bipolar OR suicide))

AND (ti:(review OR synthesis OR meta-analysis))

AND Filtered to IMSEAR database

## Ovid MEDLINE(R) ALL <1946 to September 27, 2021>

Search date: 29/09/2021

Records found: 166

1 bangladesh/ or bhutan/ or exp india/ or afghanistan/ or nepal/ or pakistan/ or sri lanka/ or Indian Ocean Islands/ (160000)

2 (Bhutan* or afghanistan* or nepal* or "sri lanka*" or maldives).ti,ab,in,kf. (39768)

3 ((Indian or Indians) not ("west indian*" or "american indian*")).ti,ab,in,kf. (137783)

4 india.ti,ab,in,kf. (597719)

5 ("south asia*" or bangladesh* or pakistan*).ti,ab,in,kf. (100070)

6 or/1-5 [S Asia] (785274)

7 exp Mental Disorders/ (1318890)

8 exp Behavioral Symptoms/ (399525)

9 Self-Injurious Behavior/ or Self Mutilation/ (11738)

10 suicide/ or suicide, attempted/ or Suicide, Completed/ (58070)

11 exp Substance-Related Disorders/px (54365)

12 exp Language Disorders/ (50536)

13 exp Intellectual Disability/ (99770)

14 (7 or 8 or 9 or 10 or 11 or 12 or 13) not (exp Elimination Disorders/ or exp Motor Disorders/) (1642855)

15 incidence/ or prevalence/ (572370)

16 epidemiologic studies/ or case-control studies/ or cohort studies/ or follow-up studies/ or exp longitudinal studies/ or prospective studies/ or retrospective studies/ or cross-sectional studies/ (2755766)

17 15 or 16 (3065776)

18 14 and 17 [Prevalence or longitudinal studies of mental illness MeSH search] (321737)

19 exp Mental Disorders/sn (568)

20 exp Behavioral Symptoms/sn (17962)

21 Self-Injurious Behavior/sn or Self Mutilation/sn (1)

22 suicide/sn or suicide, attempted/sn or Suicide, Completed/sn (16793)

23 exp Substance-Related Disorders/px and exp Substance-Related Disorders/sn (0)

24 exp Language Disorders/sn (5)

25 exp Intellectual Disability/sn (57)

26 or/19-25 [Stats studies for MeSH coded records] (18550)

27 18 or 26 [Prevalence longitudinal stats studies of mental illness MeSH search] (331975)

28 ((prevalen* or incidence* or "risk factor*" or epidemiolog*) adj4 (mental or mentally or psychiatr* or psycho* or depressi* or depressed or MDD or anxi* or phobia or phobic or agoraphobi* or dysthymi* or ADNOS)).ti,kf. (10647)

29 ((prevalen* or incidence* or "risk factor*" or epidemiolog*) adj4 (schizo* or hebephrenic* or oligophreni* or akathisi* or acathisi* or neuroleptic-induc*)).ti,kf. (816)

30 ((prevalen* or incidence* or "risk factor*" or epidemiolog*) adj4 (tardiv* adj dyskine*)).ti,kf. (132)

31 ((prevalen* or incidence* or "risk factor*" or epidemiolog*) adj4 (somatoform or somatiz* or somatis* or hysteri* or briquet or multisomat* or multi somat* or MUPs or medically unexplained)).ti,kf. (62)

32 ((prevalen* or incidence* or "risk factor*" or epidemiolog*) adj4 ((dissociative adj3 (disorder* or reaction*)) or dissociation)).ti,kf. (29)

33 ((prevalen* or incidence* or "risk factor*" or epidemiolog*) adj4 (affective* adj (disorder? or disease? or illness* or symptom?))).ti,kf. (91)

34 ((prevalen* or incidence* or "risk factor*" or epidemiolog*) adj4 (PTSD or psychological trauma or psychotrauma* or combat disorder? or war disorder?)).ti,kf. (157)

35 ((prevalen* or incidence* or "risk factor*" or epidemiolog*) adj4 ((post-trauma* or posttrauma*) adj3 (stress* or disorder?))).ti,kf. (451)

36 ((prevalen* or incidence* or "risk factor*" or epidemiolog*) adj4 ((stress or cognitive or cognition or personality or impulse control or mood or paranoid or psychotic or neurologic* or nervous or eating) adj (disorder? or illness* or disease?))).ti,kf. (1161)

37 ((prevalen* or incidence* or "risk factor*" or epidemiolog*) adj4 ((bipolar or behavio?ral or obsessive or compulsive or panic or mood or delusional) adj (disorder? or illness* or disease?))).ti,kf. (403)

38 ((prevalen* or incidence* or "risk factor*" or epidemiolog*) adj4 (trichotillomani* or OCD or obsess*-compulsi* or GAD or stress reaction? or acute stress or neuros#s or neurotic)).ti,kf. (238)

39 ((prevalen* or incidence* or "risk factor*" or epidemiolog*) adj4 (stress syndrome? or distress syndrome? or pain disorder? or dementia or alzheimer*)).ti,kf. (2544)

40 ((prevalen* or incidence* or "risk factor*" or epidemiolog*) adj4 ((substance abuse or "substance use" or drug abuse or "drug use") adj2 disorder?)).ti,kf. (123)

41 ((prevalen* or incidence* or "risk factor*" or epidemiolog*) adj4 (sleep? adj2 (disorder? or syndrome?))).ti,kf. (314)

42 ((prevalen* or incidence* or "risk factor*" or epidemiolog*) adj4 (manic or mania or amnesic syndrome? or severe stress or adjustment disorder? or behavio?ral syndrome? or habit disorder? or hallucinosis or catatonic disorder?)).ti,kf. (56)

43 ((prevalen* or incidence* or "risk factor*" or epidemiolog*) adj4 ((sexual* or gender* or psychosexual*) adj3 (dysfunction* or disorder? or dysphoria))).ti,kf. (296)

44 ((prevalen* or incidence* or "risk factor*" or epidemiolog*) adj4 (transsexual* or transvest* or fetish* or exhibitionis* or voyeuris* or p?edophil* or sadomasochis*)).ti,kf. (21)

45 ((prevalen* or incidence* or "risk factor*" or epidemiolog*) adj4 (autism or autistic or "rett syndrome?" or asperger* or dyspraxia or (developmental adj3 disorder?))).ti,kf. (640)

46 ((prevalen* or incidence* or "risk factor*" or epidemiolog*) adj4 (aphasia or dysphasia or ((speech or language) adj3 disorder?))).ti,kf. (50)

47 ((prevalen* or incidence* or "risk factor*" or epidemiolog*) adj4 ((intellectual or mental or learning) adj2 (disab* or disorder* or retard*))).ti,kf. (1014)

48 ((prevalen* or incidence* or "risk factor*" or epidemiolog*) adj4 ((emotion* or hyperkinetic or conduct or "social function*" or tic) adj2 disorder?)).ti,kf. (60)

49 ((prevalen* or incidence* or "risk factor*" or epidemiolog*) adj4 ((self or themsel* or onesel*) adj2 (aggress* or harm* or cutt* or immolat* or inflict* or injur* or mutilat* or poison* or damag* or destruct*))).ti,kf. (238)

50 ((prevalen* or incidence* or "risk factor*" or epidemiolog*) adj4 (parasuicid* or para-suicid* or suicidality or NSSI or autoaggress* or "auto aggress*" or auto-aggress)).ti,kf. (72)

51 ((prevalen* or incidence* or "risk factor*" or epidemiolog*) adj4 (suicid* adj2 (death or die* or mortality or complete or attempt* or behavio* or intent* or intend* or commit*))).ti,kf. (510)

52 ((prevalen* or incidence* or "risk factor*" or epidemiolog*) adj4 (overdos* adj2 (deliberat* or intentional or intended))).ti,kf. (4)

53 (rate? adj4 ((self or themsel* or onesel*) adj2 (aggress* or harm* or cutt* or immolat* or inflict* or injur* or mutilat* or poison* or damag* or destruct*))).ti,kf. (45)

54 (rate? adj4 (parasuicid* or para-suicid* or suicidality or NSSI or autoaggress* or "auto aggress*" or auto-aggress)).ti,kf. (9)

55 (rate? adj4 (suicid* adj2 (death or die* or mortality or complete or attempt* or behavio* or intent* or intend* or commit*))).ti,kf. (93)

56 (rate? adj4 (overdos* adj2 (deliberat* or intentional or intended))).ti,kf. (0)

57 ((case-control or cohort or follow-up or follow-on or longitudinal or prospective or retrospective or cross-sectional) adj8 (mental or mentally or psychiatr* or psycho* or depressi* or depressed or MDD or anxi* or phobia or phobic or agoraphobi* or dysthymi* or ADNOS)).ti,kf. (17682)

58 ((case-control or cohort or follow-up or follow-on or longitudinal or prospective or retrospective or cross-sectional) adj8 (schizo* or hebephrenic* or oligophreni* or akathisi* or acathisi* or neuroleptic-induc*)).ti,kf. (2513)

59 ((case-control or cohort or follow-up or follow-on or longitudinal or prospective or retrospective or cross-sectional) adj8 (tardiv* adj dyskine*)).ti,kf. (65)

60 ((case-control or cohort or follow-up or follow-on or longitudinal or prospective or retrospective or cross-sectional) adj8 (somatoform or somatiz* or somatis* or hysteri* or briquet or multisomat* or multi somat* or MUPs or medically unexplained)).ti,kf. (99)

61 ((case-control or cohort or follow-up or follow-on or longitudinal or prospective or retrospective or cross-sectional) adj8 ((dissociative adj3 (disorder* or reaction*)) or dissociation)).ti,kf. (129)

62 ((case-control or cohort or follow-up or follow-on or longitudinal or prospective or retrospective or cross-sectional) adj8 (affective* adj (disorder? or disease? or illness* or symptom?))).ti,kf. (159)

63 ((case-control or cohort or follow-up or follow-on or longitudinal or prospective or retrospective or cross-sectional) adj8 (PTSD or psychological trauma or psychotrauma* or combat disorder? or war disorder?)).ti,kf. (232)

64 ((case-control or cohort or follow-up or follow-on or longitudinal or prospective or retrospective or cross-sectional) adj8 ((post-trauma* or posttrauma*) adj3 (stress* or disorder?))).ti,kf. (564)

65 ((case-control or cohort or follow-up or follow-on or longitudinal or prospective or retrospective or cross-sectional) adj8 ((stress or cognitive or cognition or personality or impulse control or mood or paranoid or psychotic or neurologic* or nervous or eating) adj (disorder? or illness* or disease?))).ti,kf. (1533)

66 ((case-control or cohort or follow-up or follow-on or longitudinal or prospective or retrospective or cross-sectional) adj8 ((bipolar or behavio?ral or obsessive or compulsive or panic or mood or delusional) adj (disorder? or illness* or disease?))).ti,kf. (1130)

67 ((case-control or cohort or follow-up or follow-on or longitudinal or prospective or retrospective or cross-sectional) adj8 (trichotillomani* or OCD or obsess*-compulsi* or GAD or stress reaction? or acute stress or neuros#s or neurotic)).ti,kf. (406)

68 ((case-control or cohort or follow-up or follow-on or longitudinal or prospective or retrospective or cross-sectional) adj8 (stress syndrome? or distress syndrome? or pain disorder? or dementia or alzheimer*)).ti,kf. (4145)

69 ((case-control or cohort or follow-up or follow-on or longitudinal or prospective or retrospective or cross-sectional) adj8 ((substance abuse or "substance use" or drug abuse or "drug use") adj2 disorder?)).ti,kf. (187)

70 ((case-control or cohort or follow-up or follow-on or longitudinal or prospective or retrospective or cross-sectional) adj8 (sleep? adj2 (disorder? or syndrome?))).ti,kf. (268)

71 ((case-control or cohort or follow-up or follow-on or longitudinal or prospective or retrospective or cross-sectional) adj8 (manic or mania or amnesic syndrome? or severe stress or adjustment disorder? or behavio?ral syndrome? or habit disorder? or hallucinosis or catatonic disorder?)).ti,kf. (173)

72 ((case-control or cohort or follow-up or follow-on or longitudinal or prospective or retrospective or cross-sectional) adj8 ((sexual* or gender* or psychosexual*) adj3 (dysfunction* or disorder? or dysphoria))).ti,kf. (182)

73 ((case-control or cohort or follow-up or follow-on or longitudinal or prospective or retrospective or cross-sectional) adj8 (transsexual* or transvest* or fetish* or exhibitionis* or voyeuris* or p?edophil* or sadomasochis*)).ti,kf. (43)

74 ((case-control or cohort or follow-up or follow-on or longitudinal or prospective or retrospective or cross-sectional) adj8 (autism or autistic or "rett syndrome?" or asperger* or dyspraxia or (developmental adj3 disorder?))).ti,kf. (1053)

75 ((case-control or cohort or follow-up or follow-on or longitudinal or prospective or retrospective or cross-sectional) adj8 (aphasia or dysphasia or ((speech or language) adj3 disorder?))).ti,kf. (136)

76 ((case-control or cohort or follow-up or follow-on or longitudinal or prospective or retrospective or cross-sectional) adj8 ((intellectual or mental or learning) adj2 (disab* or disorder* or retard* or difficulties))).ti,kf. (889)

77 ((case-control or cohort or follow-up or follow-on or longitudinal or prospective or retrospective or cross-sectional) adj8 ((emotion* or hyperkinetic or conduct or "social function*" or tic) adj2 disorder?)).ti,kf. (95)

78 ((case-control or cohort or follow-up or follow-on or longitudinal or prospective or retrospective or cross-sectional) adj8 ((self or themsel* or onesel*) adj2 (aggress* or harm* or cutt* or immolat* or inflict* or injur* or mutilat* or poison* or damag* or destruct*))).ti,kf. (465)

79 ((case-control or cohort or follow-up or follow-on or longitudinal or prospective or retrospective or cross-sectional) adj8 (parasuicid* or para-suicid* or suicidality or NSSI or autoaggress* or "auto aggress*" or auto-aggress)).ti,kf. (129)

80 ((case-control or cohort or follow-up or follow-on or longitudinal or prospective or retrospective or cross-sectional) adj8 (suicid* adj2 (death or die* or mortality or complete or attempt* or behavio* or intent* or intend* or commit*))).ti,kf. (710)

81 ((case-control or cohort or follow-up or follow-on or longitudinal or prospective or retrospective or cross-sectional) adj8 (overdos* adj2 (deliberat* or intentional or intended))).ti,kf. (5)

82 or/28-81 [Prevalence longitudinal stats studies of mental illness TEXTWORD search] (47371)

83 meta-analysis/ or "systematic review"/ (239636)

84 (Literature review* or (systematic adj2 review*) or (narrative adj2 review*) or (critical adj2 review*) or (evidence adj2 synthesis) or scoping review* or meta-analys* or "meta analysis").ti. (306288)

85 83 or 84 [Systematic Reviews] (368922)

86 27 or 82 (349506)

87 6 and 85 and 86 (166)

## PakMediNet https://www.pakmedinet.com/

Search date: 29/09/2021

Records found: 36

Multiple searches:

• prevalence AND mental AND review

• incidence AND mental AND review

• epidemiology AND mental AND review

• "risk factors" AND mental AND review

• prevalence AND psychiatric AND review

• incidence AND psychiatric AND review

• epidemiology AND psychiatric AND review

• "risk factors" AND psychiatric AND review

• prevalence AND psychological AND review

• incidence AND psychological AND review

• epidemiology AND psychological AND review

• "risk factors" AND psychological AND review

• prevalence AND depression AND review

• incidence AND depression AND review

• epidemiology AND depression AND review

• "risk factors" AND depression AND review

• prevalence AND schizophrenia AND review

• incidence AND schizophrenia AND review

• epidemiology AND schizophrenia AND review

• "risk factors" AND schizophrenia AND review

• prevalence AND dementia AND review

• incidence AND dementia AND review

• epidemiology AND dementia AND review

• "risk factors" AND dementia AND review

· prevalence AND suicide AND review

· incidence AND suicide AND review

· epidemiology AND suicide AND review

· "risk factors" AND suicide AND review

· prevalence AND intellectual AND review

· incidence AND intellectual AND review

· epidemiology AND intellectual AND review

· "risk factors" AND intellectual AND review

· prevalence AND speech AND review

· incidence AND speech AND review

· epidemiology AND speech AND review

· "risk factors" AND speech AND review

## APA PsycInfo (Ovid) <1806 to September Week 3 2021>

Search date: 29/09/2021

Records found: 86

1 (Bhutan* or afghanistan* or nepal* or "sri lanka*" or maldives).ti,ab,in,id,lo. (8077)

2 ((Indian or Indians) not ("west indian*" or "american indian*")).ti,ab,in,id,lo. (19298)

3 india.ti,ab,in,id,lo. (46983)

4 ("south asia*" or bangladesh* or pakistan*).ti,ab,in,id,lo. (12057)

5 or/1-4 [All S Asia World Bank] (69960)

6 exp mental disorders/ (903900)

7 exp behavior disorders/ (59955)

8 posttraumatic stress/ (608)

9 psychological stress/ (9171)

10 Self-Destructive Behavior/ or exp Self-Injurious Behavior/ or exp Suicide/ (43965)

11 exp Speech Disorders/ (29753)

12 exp Neurodevelopmental Disorders/ (167013)

13 (6 or 7 or 8 or 9 or 10 or 11 or 12) not exp movement disorders/ (957284)

14 epidemiology/ (52281)

15 at risk populations/ (38989)

16 risk factors/ (87657)

17 cohort analysis/ (1547)

18 followup studies/ (12387)

19 exp longitudinal studies/ (16848)

20 or/14-19 (196362)

21 13 and 20 [Prevalence or longitudinal studies of Mental Illness Subject Headings search] (88173)

22 ((prevalen* or incidence* or "risk factor*" or epidemiolog*) adj4 (mental or mentally or psychiatr* or psycho* or depressi* or depressed or MDD or anxi* or phobia or phobic or agoraphobi* or dysthymi* or ADNOS)).ti,id. (12167)

23 ((prevalen* or incidence* or "risk factor*" or epidemiolog*) adj4 (schizo* or hebephrenic* or oligophreni* or akathisi* or acathisi* or neuroleptic-induc*)).ti,id. (981)

24 ((prevalen* or incidence* or "risk factor*" or epidemiolog*) adj4 (tardiv* adj dyskine*)).ti,id. (142)

25 ((prevalen* or incidence* or "risk factor*" or epidemiolog*) adj4 (somatoform or somatiz* or somatis* or hysteri* or briquet or multisomat* or multi somat* or MUPs or medically unexplained)).ti,id. (97)

26 ((prevalen* or incidence* or "risk factor*" or epidemiolog*) adj4 ((dissociative adj3 (disorder* or reaction*)) or dissociation)).ti,id. (43)

27 ((prevalen* or incidence* or "risk factor*" or epidemiolog*) adj4 (affective* adj (disorder? or disease? or illness* or symptom?))).ti,id. (185)

28 ((prevalen* or incidence* or "risk factor*" or epidemiolog*) adj4 (PTSD or psychological trauma or psychotrauma* or combat disorder? or war disorder?)).ti,id. (355)

29 ((prevalen* or incidence* or "risk factor*" or epidemiolog*) adj4 ((post-trauma* or posttrauma*) adj3 (stress* or disorder?))).ti,id. (407)

30 ((prevalen* or incidence* or "risk factor*" or epidemiolog*) adj4 ((stress or cognitive or cognition or personality or impulse control or mood or paranoid or psychotic or neurologic* or nervous or eating) adj (disorder? or illness* or disease?))).ti,id. (1355)

31 ((prevalen* or incidence* or "risk factor*" or epidemiolog*) adj4 ((bipolar or behavio?ral or obsessive or compulsive or panic or mood or delusional) adj (disorder? or illness* or disease?))).ti,id. (487)

32 ((prevalen* or incidence* or "risk factor*" or epidemiolog*) adj4 (trichotillomani* or OCD or obsess*-compulsi* or GAD or stress reaction? or acute stress or neuros#s or neurotic)).ti,id. (311)

33 ((prevalen* or incidence* or "risk factor*" or epidemiolog*) adj4 (stress syndrome? or distress syndrome? or pain disorder? or dementia or alzheimer*)).ti,id. (1504)

34 ((prevalen* or incidence* or "risk factor*" or epidemiolog*) adj4 ((substance abuse or "substance use" or drug abuse or "drug use") adj2 disorder?)).ti,id. (158)

35 ((prevalen* or incidence* or "risk factor*" or epidemiolog*) adj4 (sleep? adj2 (disorder? or syndrome?))).ti,id. (101)

36 ((prevalen* or incidence* or "risk factor*" or epidemiolog*) adj4 (manic or mania or amnesic syndrome? or severe stress or adjustment disorder? or behavio?ral syndrome? or habit disorder? or hallucinosis or catatonic disorder?)).ti,id. (94)

37 ((prevalen* or incidence* or "risk factor*" or epidemiolog*) adj4 ((sexual* or gender* or psychosexual*) adj3 (dysfunction* or disorder? or dysphoria))).ti,id. (166)

38 ((prevalen* or incidence* or "risk factor*" or epidemiolog*) adj4 (transsexual* or transvest* or fetish* or exhibitionis* or voyeuris* or p?edophil* or sadomasochis*)).ti,id. (27)

39 ((prevalen* or incidence* or "risk factor*" or epidemiolog*) adj4 (autism or autistic or "rett syndrome?" or asperger* or dyspraxia or (developmental adj3 disorder?))).ti,id. (483)

40 ((prevalen* or incidence* or "risk factor*" or epidemiolog*) adj4 (aphasia or dysphasia or ((speech or language) adj3 disorder?))).ti,id. (60)

41 ((prevalen* or incidence* or "risk factor*" or epidemiolog*) adj4 ((intellectual or mental or learning) adj2 (disab* or disorder* or retard* or difficulties))).ti,id. (1349)

42 ((prevalen* or incidence* or "risk factor*" or epidemiolog*) adj4 ((emotion* or hyperkinetic or conduct or "social function*" or tic) adj2 disorder?)).ti,id. (127)

43 ((prevalen* or incidence* or "risk factor*" or epidemiolog*) adj4 ((self or themsel* or onesel*) adj2 (aggress* or harm* or cutt* or immolat* or inflict* or injur* or mutilat* or poison* or damag* or destruct*))).ti,id. (227)

44 ((prevalen* or incidence* or "risk factor*" or epidemiolog*) adj4 (parasuicid* or para-suicid* or suicidality or NSSI or autoaggress* or "auto aggress*" or auto-aggress)).ti,id. (99)

45 ((prevalen* or incidence* or "risk factor*" or epidemiolog*) adj4 (suicid* adj2 (death or die* or mortality or complete or attempt* or behavio* or intent* or intend* or commit*))).ti,id. (600)

46 ((prevalen* or incidence* or "risk factor*" or epidemiolog*) adj4 (overdos* adj2 (deliberat* or intentional or intended))).ti,id. (2)

47 (rate? adj4 ((self or themsel* or onesel*) adj2 (aggress* or harm* or cutt* or immolat* or inflict* or injur* or mutilat* or poison* or damag* or destruct*))).ti,id. (55)

48 (rate? adj4 (parasuicid* or para-suicid* or suicidality or NSSI or autoaggress* or "auto aggress*" or auto-aggress)).ti,id. (24)

49 (rate? adj4 (suicid* adj2 (death or die* or mortality or complete or attempt* or behavio* or intent* or intend* or commit*))).ti,id. (207)

50 (rate? adj4 (overdos* adj2 (deliberat* or intentional or intended))).ti,id. (0)

51 ((case-control or cohort or follow-up or follow-on or longitudinal or prospective or retrospective or cross-sectional) adj8 (mental or mentally or psychiatr* or psycho* or depressi* or depressed or MDD or anxi* or phobia or phobic or agoraphobi* or dysthymi* or ADNOS)).ti,id. (14625)

52 ((case-control or cohort or follow-up or follow-on or longitudinal or prospective or retrospective or cross-sectional) adj8 (schizo* or hebephrenic* or oligophreni* or akathisi* or acathisi* or neuroleptic-induc*)).ti,id. (2389)

53 ((case-control or cohort or follow-up or follow-on or longitudinal or prospective or retrospective or cross-sectional) adj8 (tardiv* adj dyskine*)).ti,id. (51)

54 ((case-control or cohort or follow-up or follow-on or longitudinal or prospective or retrospective or cross-sectional) adj8 (somatoform or somatiz* or somatis* or hysteri* or briquet or multisomat* or multi somat* or MUPs or medically unexplained)).ti,id. (70)

55 ((case-control or cohort or follow-up or follow-on or longitudinal or prospective or retrospective or cross-sectional) adj8 ((dissociative adj3 (disorder* or reaction*)) or dissociation)).ti,id. (50)

56 ((case-control or cohort or follow-up or follow-on or longitudinal or prospective or retrospective or cross-sectional) adj8 (affective* adj (disorder? or disease? or illness* or symptom?))).ti,id. (162)

57 ((case-control or cohort or follow-up or follow-on or longitudinal or prospective or retrospective or cross-sectional) adj8 (PTSD or psychological trauma or psychotrauma* or combat disorder? or war disorder?)).ti,id. (251)

58 ((case-control or cohort or follow-up or follow-on or longitudinal or prospective or retrospective or cross-sectional) adj8 ((post-trauma* or posttrauma*) adj3 (stress* or disorder?))).ti,id. (469)

59 ((case-control or cohort or follow-up or follow-on or longitudinal or prospective or retrospective or cross-sectional) adj8 ((stress or cognitive or cognition or personality or impulse control or mood or paranoid or psychotic or neurologic* or nervous or eating) adj (disorder? or illness* or disease?))).ti,id. (1233)

60 ((case-control or cohort or follow-up or follow-on or longitudinal or prospective or retrospective or cross-sectional) adj8 ((bipolar or behavio?ral or obsessive or compulsive or panic or mood or delusional) adj (disorder? or illness* or disease?))).ti,id. (1026)

61 ((case-control or cohort or follow-up or follow-on or longitudinal or prospective or retrospective or cross-sectional) adj8 (trichotillomani* or OCD or obsess*-compulsi* or GAD or stress reaction? or acute stress or neuros#s or neurotic)).ti,id. (416)

62 ((case-control or cohort or follow-up or follow-on or longitudinal or prospective or retrospective or cross-sectional) adj8 (stress syndrome? or distress syndrome? or pain disorder? or dementia or alzheimer*)).ti,id. (2270)

63 ((case-control or cohort or follow-up or follow-on or longitudinal or prospective or retrospective or cross-sectional) adj8 ((substance abuse or "substance use" or drug abuse or "drug use") adj2 disorder?)).ti,id. (164)

64 ((case-control or cohort or follow-up or follow-on or longitudinal or prospective or retrospective or cross-sectional) adj8 (sleep? adj2 (disorder? or syndrome?))).ti,id. (88)

65 ((case-control or cohort or follow-up or follow-on or longitudinal or prospective or retrospective or cross-sectional) adj8 (manic or mania or amnesic syndrome? or severe stress or adjustment disorder? or behavio?ral syndrome? or habit disorder? or hallucinosis or catatonic disorder?)).ti,id. (185)

66 ((case-control or cohort or follow-up or follow-on or longitudinal or prospective or retrospective or cross-sectional) adj8 ((sexual* or gender* or psychosexual*) adj3 (dysfunction* or disorder? or dysphoria))).ti,id. (88)

67 ((case-control or cohort or follow-up or follow-on or longitudinal or prospective or retrospective or cross-sectional) adj8 (transsexual* or transvest* or fetish* or exhibitionis* or voyeuris* or p?edophil* or sadomasochis*)).ti,id. (44)

68 ((case-control or cohort or follow-up or follow-on or longitudinal or prospective or retrospective or cross-sectional) adj8 (autism or autistic or "rett syndrome?" or asperger* or dyspraxia or (developmental adj3 disorder?))).ti,id. (790)

69 ((case-control or cohort or follow-up or follow-on or longitudinal or prospective or retrospective or cross-sectional) adj8 (aphasia or dysphasia or ((speech or language) adj3 disorder?))).ti,id. (126)

70 ((case-control or cohort or follow-up or follow-on or longitudinal or prospective or retrospective or cross-sectional) adj8 ((intellectual or mental or learning) adj2 (disab* or disorder* or retard* or difficulties))).ti,id. (815)

71 ((case-control or cohort or follow-up or follow-on or longitudinal or prospective or retrospective or cross-sectional) adj8 ((emotion* or hyperkinetic or conduct or "social function*" or tic) adj2 disorder?)).ti,id. (119)

72 ((case-control or cohort or follow-up or follow-on or longitudinal or prospective or retrospective or cross-sectional) adj8 ((self or themsel* or onesel*) adj2 (aggress* or harm* or cutt* or immolat* or inflict* or injur* or mutilat* or poison* or damag* or destruct*))).ti,id. (348)

73 ((case-control or cohort or follow-up or follow-on or longitudinal or prospective or retrospective or cross-sectional) adj8 (parasuicid* or para-suicid* or suicidality or NSSI or autoaggress* or "auto aggress*" or auto-aggress)).ti,id. (119)

74 ((case-control or cohort or follow-up or follow-on or longitudinal or prospective or retrospective or cross-sectional) adj8 (suicid* adj2 (death or die* or mortality or complete or attempt* or behavio* or intent* or intend* or commit*))).ti,id. (579)

75 ((case-control or cohort or follow-up or follow-on or longitudinal or prospective or retrospective or cross-sectional) adj8 (overdos* adj2 (deliberat* or intentional or intended))).ti,id. (1)

76 or/22-75 [Prevalence or longitudinal studies of mental illness TEXTWORD search] (42113)

77 21 or 76 [Prevalence of Mental Illness] (114516)

78 5 and 77 [S Asia Prevalence of Mental Illness] (2066)

79 "systematic review"/ or meta analysis/ (5612)

80 (Literature review* or (systematic adj2 review*) or (narrative adj2 review*) or (critical adj2 review*) or (evidence adj2 synthesis) or scoping review* or meta-analys* or "meta analysis").ti. (52629)

81 79 or 80 (54831)

82 78 and 81 (86)

## Other Sources:

### ELDIS https://www.eldis.org/

Search date: 29/09/2021; Records found: 2

Select South Asia in Advanced Options > Focus Region.

Select Documents from Type checkboxes

Add basic searches into search box in top left corner

e.g. mental AND prevalence AND review

Search combinations of mental key terms: psychiatric, psychological, depression, schizophrenia, dementia, suicide, intellectual, speech with prevalence key terms: incidence, risk factor, epidemiology

### WHO IRIS Institutional Repository for Information Sharing https://apps.who.int/iris/

Search date: 29/09/2021; Records found: 9

Example search:

• All of IRIS: (afghanistan OR bhutan OR bangladesh OR india OR nepal OR Pakistan OR "sri lanka" OR "south asia" or maldives)

• Filter set to TI: Review AND

• Filter set to TI: Mental

Above search repeated with individual searches, replacing ‘Mental’ with alternative terms: Psychiatric, Psychological. Depression, Dementia, Schizophrenia, suicide, intellectual, speech

### World Bank Open Knowledge Repository https://openknowledge.worldbank.org/

Search date: 29/09/2021; Records found: 0

All of OKR: (afghanistan OR bhutan OR bangladesh OR india OR nepal OR Pakistan OR "sri lanka" OR "south asia" or maldives)

Filters applied:

• Ti: Mental, • Ti: Review

Alternative terms to ‘Mental’ were searched: Psychiatric, Psychological. Depression, Dementia, Schizophrenia, suicide, intellectual, speech.

This search did not include a ‘prevalence’ search concept.

# Appendix 4 – Review eligibility criteria

| **Inclusion criteria** | **Exclusion criteria** |
| --- | --- |
| 1. Systematically conducted meta-analytic and non-meta-analytic reviews | 1. Non-systematically conducted reviews, primary studies, opinions, commentaries, editorials |
| 1. Reviews reporting prevalence (or other quantitative measures like incidence) of mental disorders and intentional self-harm (ICD-10 all F-codes and X-codes 60 to 84) | 1. Reviews that do not report prevalence (or other quantitative measures) of mental disorders or intentional self-harm |
| 1. Review must be set in at least one South Asian country (Afghanistan, Bangladesh, Bhutan, India, Maldives, Nepal, Pakistan, and Sri Lanka). Multi-country studies that include one or more of these countries are eligible. | 1. Reviews set entirely outside South Asia |
| 1. All populations and settings (e.g., community, hospital, institution) are eligible | 1. Studies set in Afghanistan that only focus on the mental health of non-Afghan military personnel and veterans |

# Appendix 5 – Full-text exclusions with reasons

1. Arafat 2021 - Suicide methods in South Asia over two decades (2001-2020) The International journal of social psychiatry; Wrong outcomes
2. Baxter 2016 - Prevalence of mental, neurological, and substance use disorders in China and India: a systematic analysis; Wrong study design
3. Kupka 2003 - Rapid and non-rapid cycling bipolar disorder: A meta-analysis of clinical studies; Wrong outcomes
4. Gunnell 2007 - The global distribution of fatal pesticide self-poisoning: systematic review; Wrong outcomes
5. Mew 2017 The global burden of fatal self-poisoning with pesticides 2006-15: Systematic review; Wrong outcomes
6. Amiri 2020 - Prevalence of suicide in immigrants/refugees: a systematic review and meta-analysis; Wrong outcomes
7. Munawar 2020 - Prevalence of and Factors Contributing to Glue Sniffing in the South Asian Association for Regional Cooperation (SAARC) Region: A Scoping Review and Meta-analysis; Wrong outcomes
8. Cenat 2020 - Prevalence of symptoms of depression, anxiety, insomnia, posttraumatic stress disorder, and psychological distress among populations affected by the COVID-19 pandemic: A systematic review and meta-analysis; Wrong outcomes
9. Malhotra 2014 - Prevalence of child and adolescent psychiatric disorders in India: a systematic review and meta-analysis; Less than two databases
10. Perrin 2017 - The prevalence of diabetes-specific emotional distress in people with Type 2 diabetes: a systematic review and meta-analysis; Wrong outcomes
11. Fotheringham 2021 - Screening tools for common mental disorders in older adults in South Asia: A systematic scoping review ; Wrong outcomes
12. Chaulagain 2019 - Child and adolescent mental health problems in Nepal: A scoping review; Less than two databases
13. Charlson 2016 - The burden of mental, neurological, and substance use disorders in China and India: a systematic analysis of community representative epidemiological studies; Wrong study design
14. Rogers 2021 - Neurology and neuropsychiatry of COVID-19: A systematic review and meta-analysis of the early literature reveals frequent CNS manifestations and key emerging narratives; Wrong country
15. Xiong 2020 - Impact of COVID-19 pandemic on mental health in the general population: A systematic review; Wrong outcomes
16. Reddy 1998 - Prevalence of mental and behavioural disorders in India : a meta-analysis; Wrong study design
17. Lester 1998 - Correlates of regional suicide rates: A meta-analysis; Insufficient information
18. Munoz 2020 - Suicide and dementia: systematic review and meta-analysis; Wrong outcomes
19. Pinto 2019 - The prevalence and clinical correlates of cannabis use and cannabis use disorder among patients with bipolar disorder: A systematic review with meta-analysis and meta-regression; Wrong country
20. Zhou 2021 - The prevalence of PTSS under the influence of public health emergencies in last two decades: A systematic review and meta-analysis; Wrong country
21. Fu 2021 - Suicide rates among people with serious mental illness: a systematic review and meta-analysis; Wrong country
22. Righy 2019 - Prevalence of post-traumatic stress disorder symptoms in adult critical care survivors: a systematic review and meta-analysis; Wrong country
23. Satinsky 2021 - Systematic review and meta-analysis of depression, anxiety, and suicidal ideation among Ph. D. students; Wrong country
24. Collins 2020 - A systematic review and meta-analysis of personality disorder prevalence and patient outcomes in emergency departments; Wrong country
25. Remes 2016 - A systematic review of reviews on the prevalence of anxiety disorders in adult populations Brain and behaviour; Wrong study design
26. Bronsard 2016 - The prevalence of mental disorders among children and adolescents in the child welfare system: a systematic review and meta-analysis; Wrong country
27. Afzal 2016 - Postnatal Affective Disorders: Mapping the research in Pakistan Journal of Gender & Social Issues; Less than two databases
28. Doglioni 2021 - Depression in adults with sickle cell disease: a systematic review of the methodological issues in assessing prevalence of depression; Wrong country
29. Cucchi 2016 - Lifetime prevalence of non-suicidal self-injury in patients with eating disorders: a systematic review and meta-analysis; Wrong country
30. Falah-Hassani 2015 - Prevalence of postpartum depression among immigrant women: a systematic review and meta-analysis; Wrong country
31. Doraiswamy 2020 - Perinatal mental illness in the middle east and North Africa region-A systematic overview; Wrong study design
32. Hossain 2020 - Prevalence of mental disorders in South Asia: An umbrella review of systematic reviews and meta-analyses; Exclusion reason: Wrong study design;
33. Parra-Diaz 2021 - Does the Country Make a Difference in Impulse Control Disorders? A Systematic Review; Less than two databases
34. Singh 2021 - COVID-19 pandemic and psychological wellbeing among health care workers and general population: A systematic-review and meta-analysis of the current evidence from India; Less than two databases
35. Ferri 2005 - Global prevalence of dementia: A Delphi consensus study; Wrong study design
36. Olusanya 2018 - Developmental disabilities among children younger than 5 years in 195 countries and territories, 1990- 2016: a systematic analysis for the Global Burden of Disease Study 2016; Wrong study design
37. Babu 2021 - Gender identity disorder (GID) in adolescents and adults with differences of sex development (DSD): A systematic review and meta-analysis; Less than two databases
38. Hossain 2020 - Prevalence of comorbid psychiatric disorders among people with autism spectrum disorder: An umbrella review of systematic reviews and meta-analyses; Wrong country
39. Gudala 2013 - Diabetes mellitus and risk of dementia: A meta-analysis of prospective observational studies; Wrong outcomes
40. Srinivas 2017 - Comorbidities of epilepsy; Wrong study design
41. Hussain 2017 - Prevalence of depression among type 2 diabetes mellitus patients in India: Evidence- based systematic review and meta-analysis; Insufficient information
42. Mishra 2015 - Burgeoning burden of non-communicable diseases in Nepal: a scoping review; Less than two databases
43. JayKumar 2019 - Atmospheric variability and prevalence of common psychiatric disorders in south Asia: a meta - regressive analysis; Wrong outcomes
44. Chandra 2019 - Tuberculosis - Depression syndemic: A public health challenge; Wrong study design
45. Jahrami 2019 - Eating disorders risk among medical students: A global systematic review and meta-analysis; Wrong outcomes
46. Vos 2016 - Global, regional, and national incidence, prevalence, and years lived with disability for 310 diseases and injuries, 1990-2015: a systematic analysis for the Global Burden of Disease Study 2015; Wrong publication type
47. Joarder 2020 - A Record Review on the Health Status of Rohingya Refugees in Bangladesh; Less than two databases
48. Dhiman 2021 - A Systematic Review and Meta-analysis of Prevalence of Epilepsy, Dementia, Headache, and Parkinson Disease in India; Less than two databases
49. Malhotra 2017 - Erratum to: Prevalence of child and adolescent psychiatric disorders in India: a systematic review and meta-analysis; Insufficient information
50. Dewan 2015 - Alcohol use and alcohol use disorders in Bangladesh; Wrong study design
51. RaziaAliani 2017 - Epidemiology of Postpartum Depression in Pakistan: A Review of Literature; Insufficient information
52. Pattanayak 2012 - Relationship between child and adolescent anxiety disorders and alcohol use disorders: Synthesis of evidence and literature review between 1990-2012; Insufficient information
53. Hossain 2020 - Prevalence of mental disorders among people who are homeless: An umbrella review; Wrong study design
54. Amerio 2020 - La casa de papel: a pandemic in a pandemic; Wrong publication type
55. Gawai 2020 - Critical Review on Impact of COVID 19 and Mental Health; Wrong outcomes
56. Bej 2015 - Adolescent health problems in India: A review from 2001 to 2015; Less than two databases
57. Saddichha 2011 - Behavioral emergencies in India: would psychiatric emergency services help?; Wrong outcomes
58. Krishnamoorthy 2020 - Prevalence of psychological morbidities among general population, healthcare workers and COVID-19 patients amidst the COVID-19 pandemic: A systematic review and meta-analysis; Wrong country
59. SalimaMansoor 2017 - Trends and predictors of suicides in Pakistan; Wrong publication type
60. Knipe 2015 - Association of socio-economic position and suicide/attempted suicide in low- and middle-income countries in South and South-East Asia - a systematic review; Wrong outcomes
61. Karimi 2020 - The Migraine-Anxiety Comorbidity Among Migraineurs: A Systematic Review; Wrong country
62. Mokdad 2016 - Health in times of uncertainty in the eastern Mediterranean region, 1990-2013: a systematic analysis for the Global Burden of Disease Study 2013; Wrong outcomes
63. Faraone 2021 - The World Federation of ADHD International Consensus Statement: 208 Evidence-based conclusions about the disorder; Wong publication type
64. Ahmed 2020 - A Critical Review And Local Audit Of The Prevalence Of Mental Ill-Health In Heart Failure Patients; Wrong country
65. Kalra 2019 - Prevalence and determinants of common antenatal mental disorders among women in India: A systematic review; Wrong publication type
66. Lukmanji 2019 - The co-occurrence of epilepsy and autism: A systematic review; Wrong country;
67. Zhang 2019 - Suicidality among patients with asthma: A systematic review and meta-analysis ; Wrong country
68. Grover 2021 - Delirium Research in India: A Systematic Review; Wrong study design
69. Vos 2012 - Years lived with disability (YLDs) for 1160 sequelae of 289 diseases and injuries 1990-2010: a systematic analysis for the Global Burden of Disease Study 2010; Insufficient information
70. Fornaro 2015 - Prevalence and clinical features associated to bipolar disorder-migraine comorbidity: a systematic review; Wrong outcomes
71. Roy 2012 - Epidemiology of depression and diabetes: a systematic review; Insufficient information
72. McNojia 2016 - Challenges faced by health care professionals in providing quality of life (QOL) to alzheimer's and dementia patients: A systemic review; Wrong outcomes
73. Shukla 2021 - Psychological Health amidst COVID-19: A Review of existing literature in the Indian Context; Wrong study design
74. Thapaliya 2018 - Suicide and self-harm in Nepal: A scoping review; Wrong study design
75. Jin 2021 - Systematic review of depression and suicidality in child and adolescent (CAP) refugees; Wrong study design
76. Karunarathne 2020 - How many premature deaths from pesticide suicide have occurred since the agricultural Green Revolution; Insufficient information
77. Malik 2021 - From Heartbreak to Heart Disease: A Narrative Review on Depression as an Adjunct to Cardiovascular Disease; Wrong study design
78. Schouler-Ocak 2020 - Mental health of migrants; Wrong publication type
79. Lakhan 2020 - Prevalence of Depression, Anxiety, and Stress during COVID-19 Pandemic; Wrong study design
80. Farina 2020 - A systematic review and meta-analysis of dementia prevalence in seven developing countries: A STRiDE project Glob Public Health; Wrong publication type
81. Lang 2017 - Prevalence and determinants of undetected dementia in the community: a systematic literature review and a meta-analysis; Wrong country
82. Lansner 2020 - Development of depression in patients with oral cavity cancer: a systematic review; Wrong outcomes
83. Khambaty 2017 - Cultural aspects of anxiety disorders in India; Wrong study design
84. Peritogiannis 2021 - Research on psychotic disorders in rural areas: Recent advances and ongoing challenges; Wrong outcomes
85. Pomati 2016 - Worldwide prevalence of dementia: The challenge of missing data; Wrong publication type
86. AbuSuhaiban 2019 - Mental Health of Refugees and Torture Survivors: A Critical Review of Prevalence, Predictors, Wrong study design
87. Magyar-Russell 2011 - The prevalence of anxiety and depression in adults with implantable cardioverter defibrillators: A systematic review; Wrong country
88. Basu 2011 - Compulsive buying: An overlooked entity; Wrong outcomes
89. Morovatdar 2013 - Most common methods of suicide in Eastern Mediterranean Region of WHO: a systematic review and meta-analysis; Wrong outcomes
90. Singh 1999 - Prevalence of mental and behavioural disorders in India : a meta-analysis; Wrong publication type
91. Kaur 2019 - Diagnosis of Human Psychological Disorders using Supervised Learning and Nature-Inspired Computing Techniques: A Meta-Analysis; Wrong outcomes
92. Sharma 2021 - Preschool-onset OCD: A review of literature and clinical experience; Wrong study design
93. Lindert 2018 - [Anxiety, Depression and Posttraumatic Stress Disorder in Refugees - A Systematic Review]; Wrong population
94. Purohit 2010 - Depression in Cancer Patients: A Critical Review; Full text not available
95. McGrattan 2021 - Prevalence and risk of mild cognitive impairment in low and middle-income countries: A systematic review; Wrong outcomes

# Appendix 6 – PRISMA flow chart for primary studies included in meta-analysis

Primary studies identified from:

2 included reviews (n = 214)

Forward citation screening of 2 included reviews (n = 3)

**Identification**

Records excluded after title and abstract screening; non-CMD studies and studies not in general population (n = 186)

Records screened (n = 217)

Full-texts sought for retrieval

(n = 31)

Records not retrieved (n = 0)

**Screening**

Records excluded (n = 6):

Unclear methodology for estimating prevalence (n = 4)

Combined estimates for depression and anxiety (n = 2)

Full-texts assessed for eligibility

(n = 31)

Studies included in review

(n = 25); estimates provided for:

- Depression and Anxiety (8)
- Depression only (14)
- Anxiety only (3)

**Included**

# Appendix 7 – AMSTAR 2 quality appraisal results for included reviews

High - Zero or one non-critical weakness

Moderate - More than one non-critical weakness*

Low - One critical flaw with or without non-critical weaknesses

Critically low - More than one critical flaw with or without non-critical weaknesses

Note: Multiple non-critical weaknesses may diminish confidence in the review and it may be appropriate to move the overall appraisal down from moderate to low confidence

| **Study** | **Rating** | **1** | **2** | **3** | **4** | **5** | **6** | **7** | **8** | **9** | **10** | **11** | **12** | **13** | **14** | **15** | **16** |
| --- | --- | --- | --- | --- | --- | --- | --- | --- | --- | --- | --- | --- | --- | --- | --- | --- | --- |
| **Systematic reviews with meta-analysis (n = 25)** | | | | | | | | | | | | | | | | | |
| 2010 Barua | Critically low | Yes | No | No | No | No | No | No | No | No | No | No | No | No | No | No | Yes |
| 2014 Steel | Critically low | Yes | No | Yes | Yes | Yes | Unsure | Partial yes | Partial yes | No | No | Yes | No | No | Yes | Yes | Yes |
| 2016 Cho | Low | Yes | No | No | Yes | Unsure | Unsure | Yes | Yes | Yes | No | Yes | Unsure | Yes | No | Yes | No |
| 2017 Ranjan | Critically low | Yes | No | No | Yes | Unsure | Unsure | Partial yes | Partial yes | No | No | Yes | No | No | Yes | Yes | No |
| 2017 Upadhyay | Low | Yes | No | No | Yes | Unsure | Unsure | Partial yes | Unsure | Yes | No | Yes | Yes | Yes | Yes | Yes | Yes |
| 2018 Hussain | Moderate | Yes | Yes | Yes | Partial yes | Unsure | Yes | Partial yes | Yes | Yes | No | Yes | Yes | Yes | Unsure | Yes | No |
| 2019 Chauhan | Low | Yes | No | Unsure | Partial yes | Yes | Unsure | Partial yes | Partial yes | Unsure | No | Yes | No | No | No | No | Yes |
| 2019 Hendrickson | Low | Yes | Partial yes | Yes | Partial yes | Yes | Yes | Partial yes | Partial yes | No | No | Yes | No | No | No | No | Yes |
| 2019 Mahendran | Moderate | Yes | Yes | Yes | Partial yes | Yes | No | Partial yes | Yes | Yes | No | Yes | No | Yes | Yes | Yes | Yes |
| 2019 Pilania | High | Yes | Yes | Yes | Yes | Yes | Yes | Partial yes | Yes | Yes | No | Yes | Yes | Yes | Yes | Yes | Yes |
| 2019 Prabhu | Critically low | Yes | No | Yes | Partial yes | Unsure | Unsure | No | No | No | Yes | Unsure | No | No | Yes | No | Yes |
| 2019 Uphoff | Moderate | Yes | Yes | No | Partial yes | Yes | Unsure | Partial yes | Partial yes | Yes | No | Yes | Yes | Yes | Yes | Yes | Yes |
| 2020 Ganesan | Low | Yes | Partial yes | No | Partial yes | No | Unsure | Partial yes | Partial yes | No | No | Yes | No | No | Unsure | Yes | No |
| 2020 Khan | Moderate | Yes | Yes | Yes | Partial yes | Yes | Yes | Partial yes | Yes | Yes | No | Yes | Yes | Yes | Yes | Yes | No |
| 2020 Naveed | Moderate | Yes | Yes | Yes | Partial yes | Yes | Yes | Partial yes | Yes | Yes | No | Yes | Unsure | Yes | Unsure | Yes | Yes |
| 2021 Abraham | High/Mod | Yes | Yes | No | Yes | Yes | Yes | Yes | Yes | Yes | Yes | Yes | Unsure | Unsure | Yes | No | Yes |
| 2021 Assariparambil | Low | Yes | No | Yes | Yes | Yes | Yes | Yes | Partial yes | Yes | No | Yes | No | No | Yes | Unsure | No |
| 2021 Atif | Low | Yes | Yes | Yes | Partial yes | No | No | Partial yes | Partial yes | No | No | Yes | No | No | No | Yes | No |
| 2021 Choudhary | Critically low | Yes | No | No | Partial yes | Yes | Unsure | No | Partial yes | Yes | No | Yes | Unsure | Unsure | Yes | Yes | Yes |
| 2021 Hossain | Low | Yes | Partial yes | No | Partial yes | Yes | Yes | Partial yes | Partial yes | Unsure | No | Yes | Yes | Yes | Yes | Yes | Yes |
| 2021 Hosseinnejad | Low | Unsure | No | Yes | Yes | Unsure | Unsure | Partial yes | Partial yes | Yes | No | Unsure | No | No | Unsure | No | Yes |
| 2021 Kalra | Moderate | Yes | Yes | Yes | Partial yes | Yes | Yes | Partial yes | Yes | Yes | No | Yes | Yes | Yes | Yes | Yes | Yes |
| 2021 Patra | Low | Yes | No | Yes | Yes | Yes | Yes | Partial yes | Yes | Yes | Yes | Yes | Yes | Yes | Yes | No | Yes |
| 2021 Yadav | High/Mod | Yes | Yes | No | Yes | Yes | Yes | Partial yes | Yes | Yes | No | Yes | Yes | Yes | Unsure | Yes | Yes |
| 2021 Zuberi | Moderate | Yes | Yes | No | Yes | Yes | Yes | Partial yes | Partial yes | Yes | No | Yes | No | Unsure | Yes | Yes | Yes |
| **Systematic reviews with no meta-analysis (n = 99)** | | | | | | | | | | | | | | | | | |
| 2004 Mirza | Critically low | Yes | No | Yes | Partial yes | No | No | No | Partial yes | Yes | No | No MA | No MA | Yes | No | No MA | Yes |
| 2005 Mills | Low | Yes | Partial yes | Yes | Partial yes | Yes | Yes | Partial yes | Partial yes | No | No | No MA | No MA | No | No | No MA | No |
| 2006 Collins | Low | Yes | No | Yes | Partial yes | Unsure | Unsure | Partial yes | Partial yes | Unsure | No | No MA | No MA | Unsure | Yes | No MA | Yes |
| 2007 Lopes | Critically low | Yes | No | Yes | Partial yes | Yes | No | Partial yes | Partial yes | No | No | No MA | No MA | No | No | No MA | No |
| 2008 Mills | Critically low | Unsure | No | Unsure | Partial yes | Unsure | Unsure | No | Partial yes | No | No | No MA | No MA | No | No | No MA | No |
| 2010 Math | Critically low | Yes | No | Yes | Partial yes | No | Unsure | No | Partial yes | No | No | No | No | No | No | No | No |
| 2011 Das | Critically low | No | No | No | Partial yes | No | No | No | No | No | No | No MA | No MA | No | No | No MA | No MA |
| 2011 Maulik | Low | Yes | No | Yes | Partial yes | Yes | Yes | Partial yes | Yes | Unsure | No | Yes | Yes | Yes | Yes | Yes | Yes |
| 2012 Fisher | Critically low | Yes | No | No | Partial yes | Yes | Yes | Partial yes | Partial yes | No | No | Yes | No | No | Yes | Yes | Yes |
| 2013 Hawton | Critically low | Yes | No | No | Partial yes | Yes | Unsure | Partial yes | No | No | No | Yes | No | No | Yes | No | Yes |
| 2013 Jones | Low | Yes | No | Yes | Yes | No | No | Partial yes | Yes | Yes | No | No MA | No MA | Yes | Unsure | No MA | Unsure |
| 2013 Rajapakse | Low | Yes | No | Yes | Yes | No | No | Partial yes | Partial yes | Unsure | No | No MA | No MA | Yes | Yes | No MA | Yes |
| 2013 Udina | Critically low | Unsure | No | No | Yes | Unsure | Unsure | Yes | Yes | No | No | No MA | No MA | No | No | No MA | Yes |
| 2014 Beckwith | Critically low | Yes | No | No | Partial yes | No | No | Partial yes | No | No | No | No MA | No MA | No | Yes | No MA | No |
| 2014 De Bernier | Critically low | Yes | No | No | Partial yes | Yes | Unsure | No | Partial yes | No | No | No MA | No MA | No | No | No MA | No |
| 2014 Fuhr | Moderate | Yes | Unsure | Yes | Yes | Yes | Yes | Partial yes | Yes | Yes | No | Yes | Yes | Yes | Yes | No | Yes |
| 2014 Hossain | Critically low | Yes | No | No | Unsure | Yes | No | Partial yes | Partial yes | No | No | No MA | No MA | No | No | No MA | Yes |
| 2014 Mendenhall | Critically low | Yes | No | Yes | Partial yes | Yes | No | Partial yes | Yes | No | No | No MA | No MA | No | No | No MA | Yes |
| 2014 Medlow | Critically low | Yes | No | No | Partial yes | No | Yes | Partial yes | Partial yes | No | No | No MA | No MA | No | No | No MA | No MA |
| 2014 Pearson | Low | Unsure | Partial yes | Yes | Yes | Yes | Yes | Partial yes | No | Yes | No | No MA | No MA | Yes | No | No MA | No |
| 2014 Rane | Critically low | Yes | No | No | Yes | Yes | No | No | Partial yes | No | No | No MA | No MA | No | Yes | No MA | Yes |
| 2015 Aggarwal | Critically low | No | No | No | Yes | Yes | Unsure | No | Yes | No | No | No MA | No MA | Yes | Yes | No MA | Yes |
| 2015 Malakouti | Critically low | Yes | No | Yes | Partial yes | Yes | Yes | Partial yes | Partial yes | No | No | No MA | No MA | No | No | No MA | No |
| 2015 Norhayati | Critically low | Yes | No | No | Partial yes | Unsure | Unsure | No | Partial yes | No | No | No MA | No MA | No | No | No MA | Yes |
| 2016 Evagorou | Critically low | Yes | No | No | No | Unsure | Unsure | Partial yes | No | No | No | No MA | No MA | No | No | No MA | No |
| 2016 Jamali | Critically low | No | No | No | Partial yes | Unsure | Unsure | No | No | No | No | No MA | No MA | No | No | No MA | Yes |
| 2016 McKenzie | Low | Yes | No | Yes | Yes | Yes | Yes | Partial yes | Yes | Yes | No | No MA | No MA | No | Unsure | No MA | Unsure |
| 2016 Ottisova | Moderate | Yes | Yes | No | Yes | Yes | Yes | Partial yes | Partial yes | Yes | No | Yes | Unsure | Yes | Unsure | No | Yes |
| 2016 Sahu | Critically low | Unsure | No | No | Yes | Unsure | Unsure | No | Partial yes | No | No | No MA | No MA | No | No | No MA | Yes |
| 2017 Aggarwal | Critically low | Yes | No | Yes | Yes | Yes | Unsure | Partial yes | Partial yes | No | No | No MA | No MA | No | Unsure | No MA | Yes |
| 2017 Ahmed | Low | Yes | No | No | Yes | Yes | Unsure | Partial yes | Partial yes | Unsure | No | No MA | No MA | Unsure | Yes | No MA | Yes |
| 2017 Dennis | Low | Yes | No | No | Yes | No | No | Partial yes | Partial yes | Yes | No | Yes | Yes | Yes | Unsure | No | Yes |
| 2017 Hossain | Critically low | Yes | No | No | Partial yes | Yes | No | Partial yes | Partial yes | No | No | No MA | No MA | No | No | No MA | Yes |
| 2017 Naskar | Critically low | Yes | No | No | Partial yes | Yes | Yes | Partial yes | Partial yes | No | No | No MA | No MA | No | No | No MA | Yes |
| 2017 Salmanian | Low | Yes | Yes | No | Yes | Unsure | Yes | No | Yes | Yes | No | No MA | No MA | No | No | No MA | Yes |
| 2017 Singh | Critically low | No | No | No | Yes | Unsure | Unsure | No | No | No | No | No MA | No MA | No | No | No MA | Yes |
| 2017 Woody | Critically low | Yes | No | No | Yes | No | No | No | No | Yes | No | Yes | Yes | No | Yes | Yes | Yes |
| 2018 Halim | Critically low | Yes | No | No | Partial yes | Yes | Unsure | Partial yes | Partial yes | No | No | No MA | No MA | No | No | No MA | No MA |
| 2018 Hunt | Low | Yes | No | Yes | Partial yes | Unsure | No | Partial yes | Yes | Yes | No | Yes | Yes | Yes | Yes | Yes | Yes |
| 2018 Jha | Low | Yes | No | Yes | Partial yes | No | No | Partial yes | Yes | Yes | No | Unsure | No | No | No | No | Yes |
| 2018 Morina (a) | Critically low | Yes | No | Yes | Partial yes | Yes | Yes | Partial yes | Yes | No | No | No MA | No MA | No | No | No MA | No MA |
| 2018 Morina (b) | Moderate | Yes | Yes | Yes | Partial yes | Yes | Unsure | Partial yes | Yes | Yes | Yes | Yes | No | No | Yes | Yes | No |
| 2018 Shorey | Moderate | Yes | Yes | No | Yes | Yes | Unsure | Partial yes | Partial yes | Yes | No | Yes | No | No | Yes | Yes | Yes |
| 2018 Thapa | Critically low | Unsure | No | No | Yes | Unsure | No | No | Yes | Yes | No | No MA | No MA | Yes | Yes | No MA | Unsure |
| 2019 Arafat | Critically low | No | No | No | Partial yes | No | No | Partial yes | No | No | No | No MA | No MA | No | No | No MA | Yes |
| 2019 Bhagavathula | Critically low | Yes | No | Unsure | Partial yes | Yes | Yes | Yes | Partial yes | No | No | Yes | Unsure | Unsure | Yes | Yes | Yes |
| 2019 Gilmoor | Critically low | Yes | No | No | Partial yes | Unsure | Unsure | Partial yes | Partial yes | No | No | No MA | No MA | No | No | No MA | No MA |
| 2019 Knipe | High/Mod | Yes | Yes | Yes | Yes | Yes | Yes | Yes | Yes | Yes | No | Yes | Yes | Yes | Yes | No | Yes |
| 2019 Mytton | Critically low | Yes | No | Yes | Partial yes | Yes | Yes | Partial yes | Partial yes | No | Yes | No MA | No MA | No | Yes | No MA | Yes |
| 2019 Tay | Critically low | Unsure | No | No | Yes | No | No | No | No | No | No | No MA | No MA | No | No | No MA | Yes |
| 2020 Abate | Moderate | Yes | Partial yes | No | Yes | Yes | Yes | Yes | Partial yes | Yes | No | Yes | Yes | Yes | Yes | Yes | Yes |
| 2020 Akhtar | Moderate | Yes | Yes | No | Partial yes | Yes | Yes | Partial yes | Yes | Yes | No | Yes | Yes | Yes | Yes | Yes | Yes |
| 2020 Banerjee | Critically low | Yes | No | No | Partial yes | Yes | Yes | Partial yes | Partial yes | No | No | No MA | No MA | No | No | No MA | No MA |
| 2020 Blackmore | Moderate | Yes | Yes | Unsure | Yes | Yes | Yes | Partial yes | Partial yes | Yes | No | Yes | Yes | Yes | Yes | Yes | Yes |
| 2020 Dadi | Moderate | Yes | Yes | Yes | Partial yes | Unsure | Unsure | Yes | Partial yes | Yes | No | Yes | Yes | Yes | Yes | Yes | Unsure |
| 2020 Devarapalli | Critically low | Yes | No | No | Partial yes | Yes | Unsure | No | Partial yes | No | No | No MA | No MA | No | No | No MA | Yes |
| 2020 Dua | Critically low | Yes | No | No | Partial yes | No | No | Partial yes | No | No | No | No MA | No MA | No | Yes | No MA | Yes |
| 2020 Gilan | Low | Yes | Partial yes | No | Yes | Yes | Yes | Partial yes | Yes | No | No | No MA | No MA | No | No | No MA | Yes |
| 2020 Hunt | Critically low | Yes | No | Yes | Partial yes | No | Yes | No | Yes | Yes | No | Yes | Yes | Yes | Yes | Yes | Yes |
| 2020 Junaid | Low | Yes | No | Yes | Partial yes | Yes | No | Partial yes | Yes | Yes | No | Yes | Yes | Unsure | Yes | No | No |
| 2020 Karimi | Low | Unsure | No | Yes | Partial yes | Yes | No | Partial yes | Yes | Yes | No | No MA | No MA | Yes | No | No MA | No MA |
| 2020 Lasheras | Low | Yes | No | Yes | Partial yes | Unsure | No | Partial yes | Partial yes | Yes | No | Yes | No | No | Unsure | Yes | Yes |
| 2020 Liu | High/Mod | Yes | Yes | Yes | Yes | Yes | No | Partial yes | Yes | Yes | Yes | Yes | Unsure | No | Yes | Yes | Yes |
| 2020 Qiu | Low | Yes | No | Yes | Partial yes | Yes | Unsure | Partial yes | Yes | Yes | No | Yes | Yes | Yes | Yes | Yes | Yes |
| 2020 Rahele | Critically low | Yes | No | Unsure | Partial yes | Unsure | Yes | No | Partial yes | Yes | No | No MA | No MA | No | Unsure | No MA | No MA |
| 2020 Winsper | High/Mod | Yes | Yes | No | Yes | Yes | No | Yes | Yes | Yes | Yes | Yes | Yes | Yes | Yes | Yes | Yes |
| 2020 Yan | Moderate | Yes | Yes | Yes | Yes | Yes | Yes | Partial yes | Partial yes | Yes | No | Yes | Yes | Yes | No | No | Yes |
| 2021 Al Falasi | Low | Yes | No | Yes | Partial yes | Yes | Unsure | Partial yes | Partial yes | Yes | No | Yes | No | No | Yes | No | Yes |
| 2021 Al Mamun | Critically low | Yes | No | Yes | Partial yes | Unsure | Unsure | Yes | Yes | No | No | No MA | No MA | No | Yes | No MA | No MA |
| 2021 Amiri | Critically low | Yes | No | Unsure | Partial yes | Unsure | Unsure | Partial yes | Yes | No | No | No | Unsure | Yes | Yes | No | No |
| 2021 Dong | Moderate | Yes | Partial yes | Unsure | Yes | Yes | Yes | Partial yes | Partial yes | Yes | No | Yes | Unsure | Yes | Yes | Yes | Yes |
| 2021 Dutta | Mod/Low | Yes | Yes | Yes | Partial yes | Yes | Yes | Partial yes | Partial yes | Unsure | No | Yes | Yes | No | Yes | No | Yes |
| 2021 Fellmeth | High/Mod | Yes | Yes | No | Yes | Yes | Yes | Partial yes | Yes | Yes | Yes | Yes | Yes | Yes | Unsure | Yes | Yes |
| 2021 Ghazanfarpour | Critically low | No | No | No | Partial yes | Yes | No | Partial yes | Yes | No | No | Yes | No | No | No | Yes | Yes |
| 2021 Hosen | Critically low | Yes | No | No | Partial yes | No | Yes | Partial yes | Partial yes | No | No | No MA | No MA | No | No | No MA | No MA |
| 2021 Jephtha | Critically low | Yes | No | Unsure | Unsure | No | No | No | Partial yes | No | No | No | No | No | No | No | Yes |
| 2021 Kar | Critically low | Unsure | No | Unsure | Partial yes | No | Yes | Unsure | No | Yes | No | No MA | No MA | No | No | No MA | No MA |
| 2021 Liu | Mod/High | Yes | Yes | Yes | Partial yes | Yes | Yes | Partial yes | Yes | Yes | No | Yes | Yes | Yes | Yes | Yes | No |
| 2021 Mahadevan | Mod/High | Yes | Partial yes | Yes | Yes | Yes | Yes | Partial yes | Yes | Yes | Yes | Yes | No | No | Yes | No | Yes |
| 2021 Mahmud | Moderate | Yes | Partial yes | Yes | Partial yes | Yes | Yes | Partial yes | Yes | Yes | No | Yes | Yes | No | No | Yes | Yes |
| 2021 Mamun | Critically low | Yes | No | Yes | Partial yes | No | No | Partial yes | Partial yes | No | No | No MA | No MA | No | No | No MA | No MA |
| 2021 Mohammad | High | Yes | Yes | Yes | Yes | Yes | Yes | Partial yes | Yes | Yes | No | Yes | Yes | Yes | Yes | Yes | Yes |
| 2021 Necho | Low | Yes | No | No | Yes | Unsure | Yes | Partial yes | Partial yes | Yes | No | Yes | No | Yes | No | Yes | Yes |
| 2021 Panda | Low | Unsure | No | Yes | Partial yes | Yes | Yes | Partial yes | Partial yes | Yes | No | Yes | Unsure | Unsure | Yes | Unsure | No |
| 2021 Santabarbara | Moderate | Yes | Partial yes | No | Yes | Yes | Unsure | Partial yes | Yes | Yes | No | Yes | Yes | Unsure | Yes | Yes | Yes |
| 2021 Silva | Low | Yes | Yes | Unsure | Partial yes | Yes | Unsure | Partial yes | Partial yes | No | No | No MA | No MA | No | No | No MA | Yes |
| 2021 Vanderkruik | Moderate | Yes | Partial yes | Unsure | Yes | Yes | Yes | Partial yes | Partial yes | Yes | No | No MA | No MA | Yes | No | No MA | Yes |
| 2021 Wang | Critically low | Yes | Unsure | No | Partial yes | Yes | Unsure | Partial yes | No | No | No | Yes | No | No | No | Yes | Yes |
| 2009 Klainin | Critically low | Yes | No | Yes | Partial yes | No | No | Partial yes | Yes | No | No | No MA | No MA | No | No | No MA | Yes |
| 2013 Nadkarni | Critically low | Yes | No | No | Partial yes | No | No | Partial yes | Partial yes | No | No | No MA | No MA | No | No | No | No |
| 2013 Newman | Critically low | Unsure | No | Yes | Partial yes | No | No | Partial yes | Partial yes | No | No | No MA | No MA | No | Yes | No MA | Yes |
| 2014 Jordans | Moderate | Yes | Partial yes | Yes | Yes | Yes | Unsure | Partial yes | Yes | Yes | No | No MA | No MA | Yes | Yes | No MA | Yes |
| 2017 Balhara | Critically low | No | No | No | Partial yes | Unsure | Unsure | No | Partial yes | No | No | No MA | No MA | No | No | No MA | Yes |
| 2017 Kuppili | Critically low | Unsure | No | Yes | Partial yes | No | No | Partial yes | Yes | No | No | No MA | No MA | No | No | No MA | Yes |
| 2018 Shekhani | Critically low | Yes | No | No | Yes | Yes | No | Partial yes | Partial yes | No | No | No MA | No MA | No | No | No MA | Yes |
| 2019 Somrongthong | Critically low | Unsure | No | No | Partial yes | Yes | No | No | Partial yes | No | No | No MA | No MA | No | No | No MA | Yes |
| 2019 Vaidyanathan | Critically low | No | No | Yes | Yes | No | No | No | Partial yes | No | No | No MA | No MA | No | No | No MA | Yes |
| 2020 Kalra | Critically low | Yes | No | Yes | Yes | Yes | Yes | Partial yes | Yes | No | No | No MA | No MA | No | No | No MA | No |
| 2020 Rensburg | Critically low | Unsure | No | Unsure | Partial yes | Yes | No | No | Unsure | No | No | No MA | No MA | No | No | No MA | Yes |

#

# Appendix 8 – Distribution of reviews by type and mental disorders (ICD-10 codes)

| **Mental disorder and population** | **Systematic reviews with meta-analysis (n = 25)** | **Systematic reviews with no pooled estimates (n = 99)** |
| --- | --- | --- |
| **Any mental disorder** | | |
| General and clinical | Multi-country 1 | Bangladesh 1, India 1 |
| People with HIV/AIDS | - | Multi-country 1 |
| People with suicidal behaviour | Multi-country 1 | Multi-country 1 |
| **Common mental disorders (CMDs; largely combination of mood and anxiety disorders)** | | |
| Children and adolescents | - | Multi-country 1 (COVID-19) |
| General and clinical | Multi-country 2 | Multi-country 1 (COVID-19), Pakistan 1 |
| HCWs | - | India 1 (COVID-19) |
| Perinatal women | India 1 | Multi-country 3 (1 COVID-19) |
| Teachers | - | India 1 (COVID-19) |
| **F01-F09: Mental disorders due to known physiological conditions** | | |
| General and clinical | - | India 1 |
| Older people | India 1 | India 1 |
| **F10-F19: Mental and behavioural disorders due to psychoactive substance use** | | |
| General and clinical | Multi-country 2 | Multi-country 1, India 1 |
| Older people | - | India 1 |
| People with HIV/AIDS | - | Multi-country 1 |
| People with psychosis | - | Multi-country 1 |
| People with tuberculosis | - | Multi-country 1 |
| Tribal population | - | India 1 |
| **F20-F29: Schizophrenia, schizotypal, delusional, and other non-mood psychotic disorders** | | |
| General and clinical | - | India 1 |
| People with HIV/AIDS | - | Multi-country 1 |
| People with SUD | - | India 1 |
| **F30-F39: Mood [affective] disorders** | | |
| Children and adolescents | - | India 2; 1 in homeless |
| General and clinical | Multi-country 3 (1 COVID-19) | Multi-country 2 (1 COVID-19), Bangladesh 2 (1 COVID-19), India 1 |
| HCWs | Multi 1 (COVID-19), Pakistan 1 | Multi-country 1 (COVID-19), India 1 (COVID-19) |
| Older people | Multi-country 1, India 2 | Nepal 1 |
| Perinatal women | Multi-country 2, India 1, Pakistan 1 | Multi-country 11 (2 COVID-19), Bangladesh 1 in adolescents, India 1, Sri Lanka 1 (COVID-19) |
| Peri-menopausal women | India 1 | - |
| People with COVID-19 | - | India 1 |
| People with Dhat | - | Multi-country 1 |
| People with HIV/AIDS | - | Multi-country 1, India 1 |
| Amputees | - | India 1 |
| People with NCD | Multi-country 1, India 1 | Multi-country 2, India = 1 |
| People who have self-harmed | - | India 1 |
| Stroke survivors | India 1 | Multi-country 1 |
| People with SUD | India 1 | India 1 |
| People with tuberculosis | - | Multi-country 1 |
| Refugee/IDP | - | Multi-country 2, Bangladesh 1, India 1, Nepal 2 |
| Victims of human trafficking | - | Nepal 1 |
| Tribal population | - | India 1 |
| Students | Pakistan 1 | Multi-country 1, Bangladesh 2 (COVID-19) |
| People with suicidal behaviour | - | Multi-country 1 |
| **F40-F48: Anxiety, dissociative, stress-related, somatoform and other nonpsychotic mental disorders** | | |
| Children and adolescents | - | India = 1 |
| General and clinical population | Multi-country 3 (1 COVID-19), Pakistan 1 | Multi-country 2 (COVID-19), Bangladesh 1 (COVID-19), India 4 (2 COVID-19) |
| Adult males | - | Multi-country = 1 (Dhat) |
| HCWs | Multi-country 1 (COVID-19) | Multi-country 1 (COVID-19), India 1 (COVID-19) |
| Older people | - | Nepal = 1 |
| Perinatal women | - | Multi-country 4 (2 COVID-19), Bangladesh 1, Sri Lanka 1 (COVID-19) |
| People with COVID-19 | - | India 1 |
| People with Dhat | - | Multi-country 1 |
| People with HIV/AIDS | - | Multi-country 1, India 1 |
| Amputees | - | India 1 |
| People with migraine | - | India 1 |
| People with NCD | Multi-country 1 | - |
| Stroke survivors | - | Multi-country 1 |
| People with SUD | India 1 | - |
| People with tuberculosis | - | Multi-country 1 |
| Pre-operative patients | - | Multi-country 1 |
| Refugees/IDP | - | Multi-country 2, Bangladesh 1, India 1, Nepal 2 |
| Students | - | Bangladesh 2 (COVID-19), India 1 (COVID-19) |
| Victims of human trafficking | - | Nepal 1 |
| Tribal population | - | India 1 |
| **F50-F59: Behavioural syndromes associated with physiological disturbances and physical factors** | | |
| General and clinical | - | India 1 |
| Tribal population | - | India 1 |
| **F60-F69: Disorders of adult personality and behaviour** | | |
| General and clinical | - | Multi-country 1, India 2 |
| **F70-F79: Intellectual disabilities** | | |
| General and clinical | - | Multi-country 1, India 2 |
| **F80-F89: Pervasive and specific developmental disorders** | | |
| Children and adolescents | India 1 | Multi-country 2, Pakistan 1 |
| **F90-F98: Behavioural and emotional disorders with onset usually occurring in childhood and adolescence** | | |
| General and clinical | - | India 1 |
| Children and adolescents | - | Multi-country 1, Afghanistan 1, Bangladesh 1, India 2, Pakistan 1 |
| **F99-F99: Unspecified mental disorder** | | |
| General and clinical | - | - |
| **X60-X84: Intentional self-harm** | | |
| Children and adolescents | India 1 | India 2 |
| General and clinical | Multi-country 1 | Multi-country 1, Bangladesh 2 (1 COVID-19), India 2, Nepal 1, Pakistan 2, Sri Lanka 2 |
| Perinatal women | - | Multi-country 3 |
| People with HIV/AIDS | - | Multi-country 1, India 1 |
| People who have self-harmed | - | Multi-country 1, Sri Lanka 1 |
| Sex worker population | - | India 1 |
| Tribal population | - | India 1 |

Abbreviations: AUD – Alcohol Use Disorder, CMD – Common Mental Disorder, HCW – Health Care Worker, IDP – Internally Displaced Population, NCD – Non-Communicable Disease, SUD – Substance Use Disorder.

# Appendix 9 – Additional forest plots and funnel plots

## Depression – subgroup, country


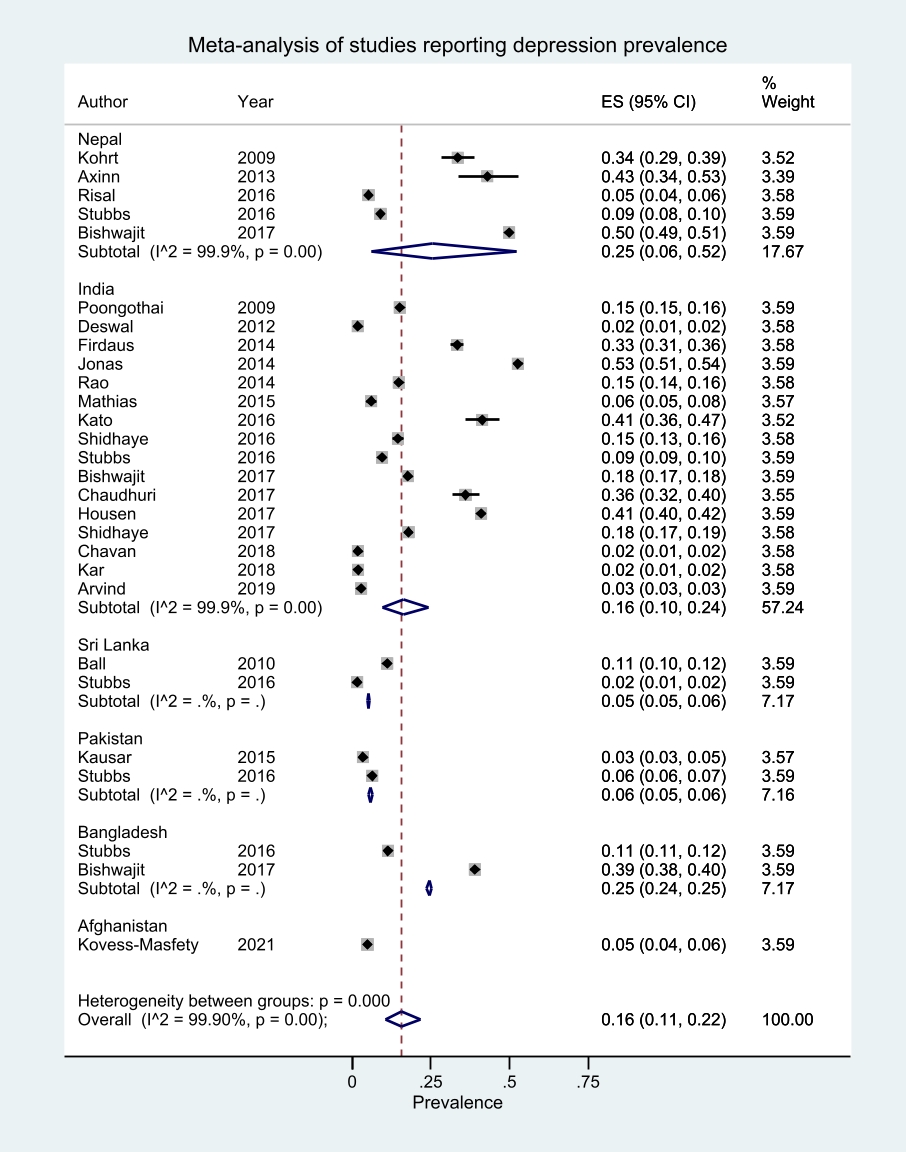


## Depression – subgroup, assessment tool


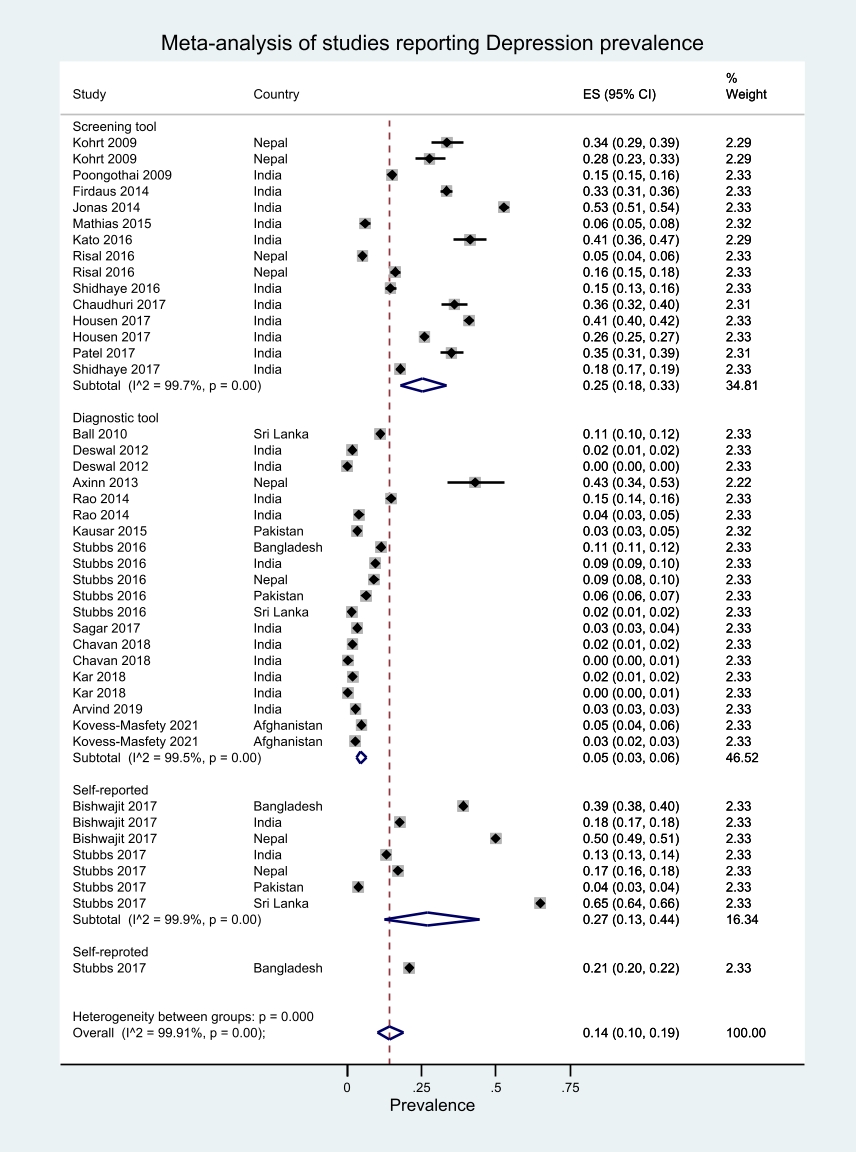


## Anxiety – subgroup, country


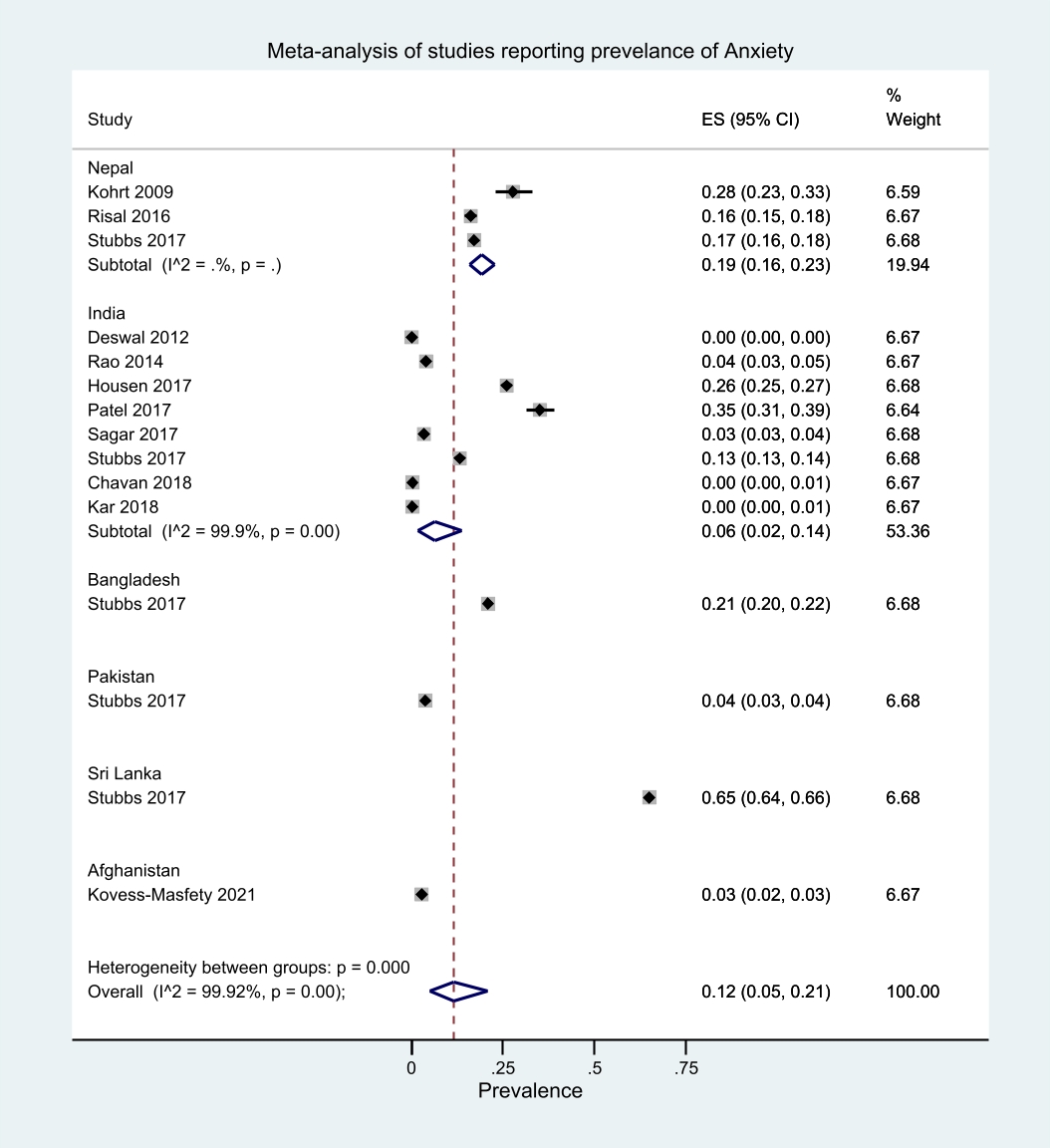


## Anxiety – subgroup, assessment tool


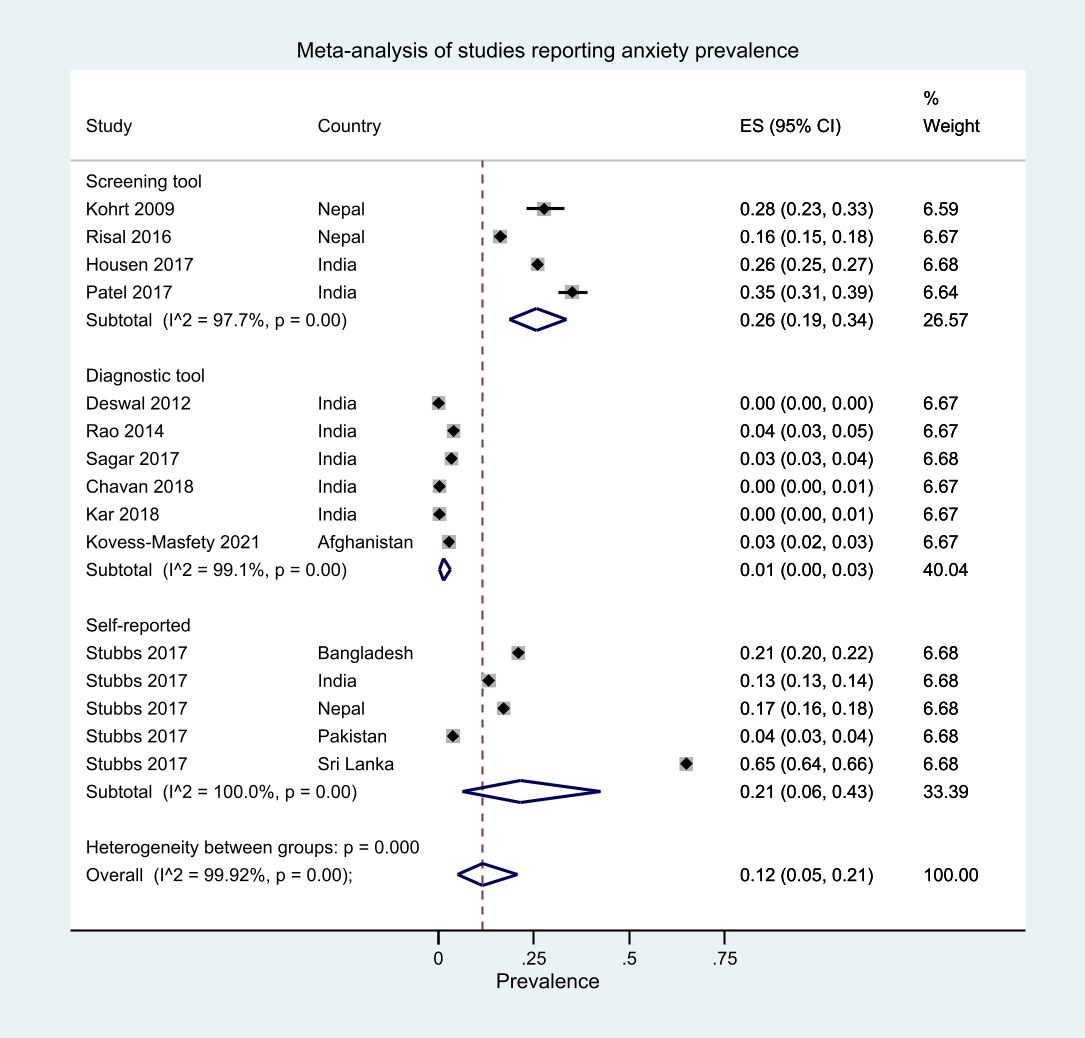


## Funnel plots – depression and anxiety


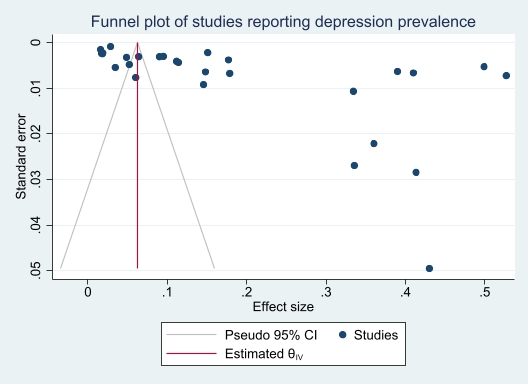


Egger’s test: H0: beta1 = 0; no small-study effects

beta1 = 9.10, SE of beta1 = 2.601, z = 3.50, Prob > |z| = 0.0005


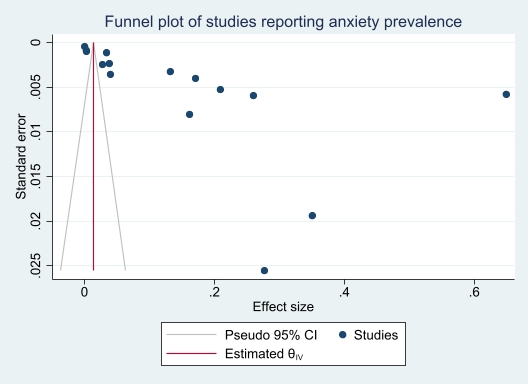


Egger’s test: H0: beta1 = 0; no small-study effects

beta1 = 3.30, SE of beta1 = 3.747, z = 0.88, Prob > |z| = 0.3779

## CMDs with older studies excluded from main analysis


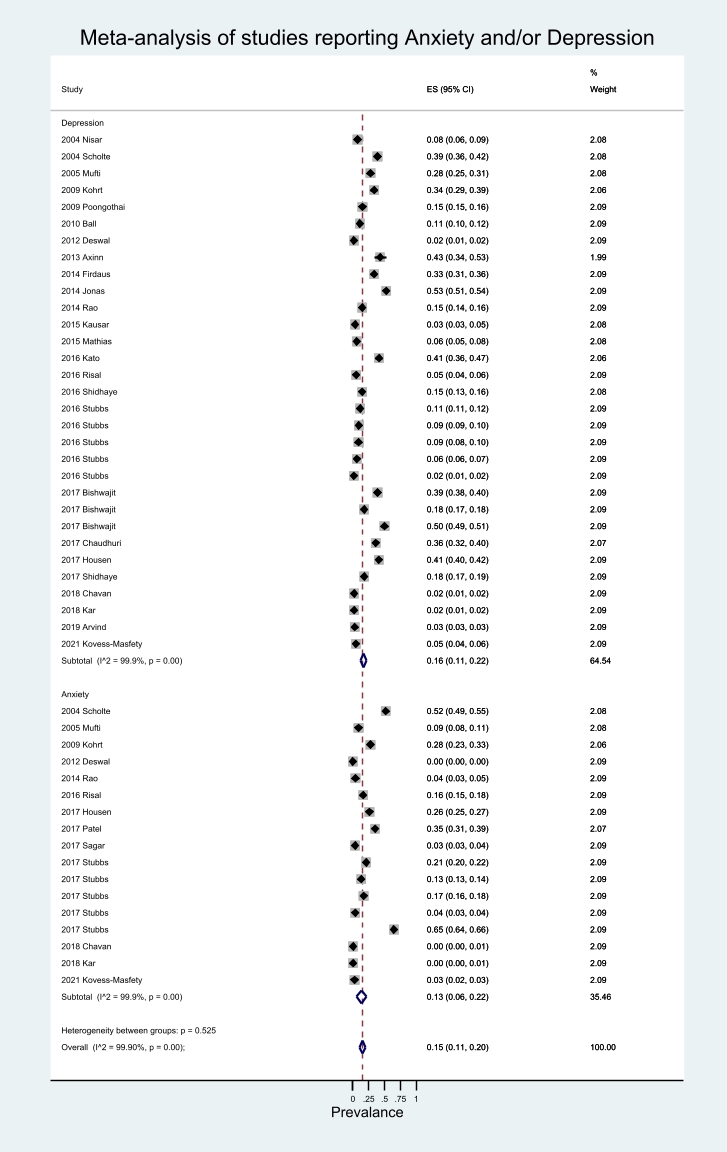

Supplement: Vidyasagaran et al. supplementary material [file S2054425123000729sup001.docx]
